# 2-Phenyl-tetrahydropyrimidine-4(1*H*)-ones – cyclic benzaldehyde aminals as precursors for functionalised β^2^-amino acids

**DOI:** 10.3762/bjoc.5.43

**Published:** 2009-09-14

**Authors:** Markus Nahrwold, Arvydas Stončius, Anna Penner, Beate Neumann, Hans-Georg Stammler, Norbert Sewald

**Affiliations:** 1 Bielefeld University, Department of Chemistry, Organic and Bioorganic Chemistry, Universitätsstr. 25, 33615 Bielefeld, Germany; 2 Bielefeld University, Department of Chemistry, Inorganic Chemistry, Universitätsstr. 25, 33615 Bielefeld, Germany

**Keywords:** β^2^-amino acids, cyclocondensation, diastereoselective alkylation, *N*,*N*-acetals, peptidomimetics, ring opening, self-regeneration of stereocentres (SRS)

## Abstract

Novel procedures have been developed to condense benzaldehyde effectively with β-amino acid amides to cyclic benzyl aminals. Double carbamate protection of the heterocycle resulted in fully protected chiral β-alanine derivatives. These serve as universal precursors for the asymmetric synthesis of functionalised β^2^-amino acids containing acid-labile protected side chains. Diastereoselective alkylation of the tetrahydropyrimidinone is followed by a chemoselective two step degradation of the heterocycle to release the free β^2^-amino acid. In the course of this study, an L-asparagine derivative was condensed with benzaldehyde and subsequently converted to orthogonally protected (*R*)-β^2^-homoaspartate.

## Introduction

Monosubstituted β-amino carboxylic acids can be classified according to their substitution pattern into α-substituted “β^2^-amino acids” and β-substituted “β^3^-amino acids” [1]. Oligomers of these β-amino acids are called “β-peptides” and tend to form distinct and stable secondary structures even at a very short chain lengths [2–3]. β-Peptides are metabolically stable peptidomimetics that have proved to be inert to enzymatic proteolysis both in vitro [4–5] and in vivo [6]. As a first biologically active example, the β-tetrapeptide Ac-β^3^hThr-β^2^hLys-β^3^hTrp-β^3^hPhe-NH_2_ was found to bind to a human somatostatin receptor with nanomolar affinity [7–9]. Despite their interesting properties, β^2^-amino acids in particular occur only rarely in nature. Several peptidic natural products contain 3-amino-2-methylpropionic acid (β^2^-homoalanine) as a building block [10]. Examples are cryptophycin-1 (a highly cytotoxic depsipeptide produced by cyanobacteria *Nostoc* sp. GSV224 and ATCC53789) [11–14] as well as a class of lipopeptides isolated from various fungi, comprising topostatin (a topoisomerase I and II inhibitor) [15], YM-170320 (an inhibitor of ergosterol biosynthesis) [16], and fusaristatins A and B [17].

β^3^-Homoamino acids can be synthesised by Arndt–Eistert homologation of the corresponding proteinogenic α-amino acids [18–20]. Contrary to that, no procedure is known yet to enantiospecifically convert α-amino acids into their β^2^-homologues – although this goal has been achieved diastereoselectively under auxiliary control [21–22]. A vast number of synthetic approaches to β^2^-(homo)amino acids have been developed so far, but the majority of these procedures are limited to α-alkyl substituted β^2^-amino acids – mostly due to harsh conditions of auxiliary cleavage or limited substrate tolerance [10,23–24]. Diastereoselective total synthesis starting from *N*-acylated Evans’ type auxiliaries turned out to be the only universal route to β^2^-analogues of the 20 most common proteinogenic α-amino acids. (For a recent overview on β^2^-amino acid syntheses see ref. [23].)

**Scheme 1:**
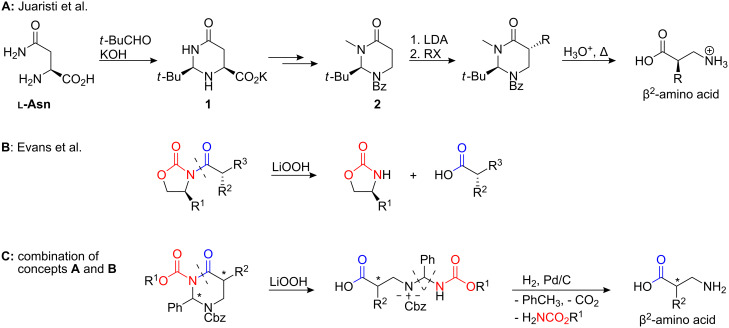
Selectively cleavable β^2^-amino acid precursors: Structures and reactivities of 2-*tert*-butyl-tetrahydropyrimidine-4(1*H*)-ones (**A**), *N*-acylated Evans auxiliaries (**B**), and novel Cbz-protected 2-phenyl-tetrahydropyrimidine-4(1*H*)-ones (**C**).

The tetrahydropyrimidine-4(*H*)-one **2** developed by Juaristi et al. [25–26] and Konopelski et al. [27–28] represents a chiral cyclic β-alanine derivative that serves as a straightforward β^2^-amino acid precursor (Scheme 1A). Following the principle of ***s****elf* ***r****egeneration of* ***s****tereogenic centres* (SRS) proposed by Seebach [29], condensation of L-asparagine and pivalaldehyde yields *N*,*N*′-acetal **1**, which is converted to **2** by subsequent oxidative decarboxylation [25,28,30–33], hydrogenation of the resulting olefinic double bond [25–27,31,34–35], and final *N*,*N*′-protection. Compound **2** proved to be a versatile β^2^- and β^2,2^-amino acid precursor. Monoalkylation of **2** takes place in high yields and with high *trans*-selectivity. Inversion of the introduced stereogenic centre via diastereoselective protonation [34] as well as α,α-dialkylation [36] both proceed smoothly. However, complete hydrolysis of the alkylated *N*,*N*-acetal requires refluxing in concentrated aqueous mineral acid. Therefore, precursor **2** can only be applied for the synthesis of target compounds without acid labile functional groups. Seebach et al. partly circumvented this problem by cleaving α-alkylated imino esters of tetrahydropyrimidine-4-ones to corresponding β^2^-amino acid methyl esters under markedly milder acidic conditions [37].

Nevertheless, a derivative of **2** being cleavable under neutral or slightly basic conditions was still unknown. Such a precursor would allow the synthesis of β^2^-amino acids containing e.g. *tert*-butyl protected side chain functions. Amino acids protected like that are particularly suitable for solid phase peptide synthesis and are thus highly desirable. Our novel ring cleavage concept includes the protection of both ring-nitrogen atoms as carbamates (Scheme 1C). Similar to the cleavage conditions of Evans’ auxiliaries (Scheme 1B) [38], the tetrahydropyrimidinone ring could now be regioselectively opened by treatment with lithiumhydroperoxide. If the original C^2^-*tert*-butyl function was additionally substituted by a phenyl group through replacement of pivalaldehyde by benzaldehyde, this structural modification would facilitate a final release of the β^2^-amino acid by hydrogenolysis of all remaining benzyl-type *N*-protective groups.

## Results and Discussion

Cyclocondensation of benzaldehyde and β-amino acid amides to 2-phenyl-tetrahydropyrimidine-4(1*H*)-ones is problematic, since acyclic resonance stabilised Schiff bases are preferentially formed. Consequently, one pot cyclisation protocols employing conditions such as KOH in protic solvents deliver the desired six-membered ring only in unsatisfactory yields [30,39]. An alternative approach to cyclise benzaldehyde-derived imines consists of a 4-DMAP catalysed *N*-acylation reaction with acyl chlorides and usually affords the target compounds in moderate yields [39–40].

**Scheme 2:**
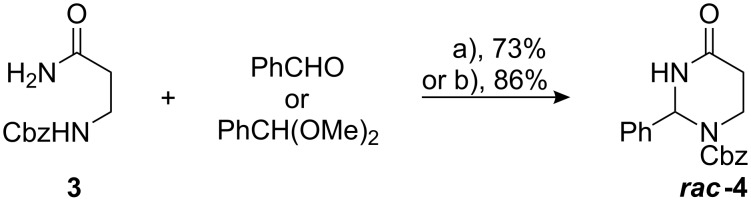
Cyclocondensation of Cbz-^β^Ala-NH_2_ (**3**) and benzaldehyde. a) [*p*-TsOH], toluene, reflux, Dean–Stark trap, 3 h; b) BF_3_·Et_2_O (2.0 equiv), CH_2_Cl_2_, rt, 16 h.

We chose *N*^α^-Cbz-protected β-alanine amide (**3**) as starting material for the synthesis of the 2-phenyltetrahydropyrimidine-4(1*H*)-one ***rac*-4** to circumvent the difficulty of cyclising stabilised benzyl imines. Cyclocondensation was successfully carried out under two different reaction conditions (Scheme 2). On the one hand, **3** was reacted with benzaldehyde or with the corresponding dimethylacetal in refluxing toluene in the presence of a catalytic amount of *p*-TsOH. The water or methanol formed during condensation was distilled azeotropically from the reaction mixture. An even more effective method turned out to be the BF_3_·Et_2_O-mediated condensation of **3** with benzaldehyde or benzaldehyde dimethylacetal. Since the Lewis acid serves both as activating agent and as irreversible water/methanol trapping agent, two equivalents of it are necessary to drive the reaction to completion. The 2-phenyl-tetrahydropyrimidine-4-one ***rac*-4** obtained by either of the two methods can be purified by crystallisation or chromatography. Compound **4** proved to be stable to air moisture at room temperature. Despite the success in condensing **3** and benzaldehyde (dimethylacetal), all attempts to condense **3** with closely related acetophenone dimethylketal did not lead to any cyclisation product.

**Scheme 3:**
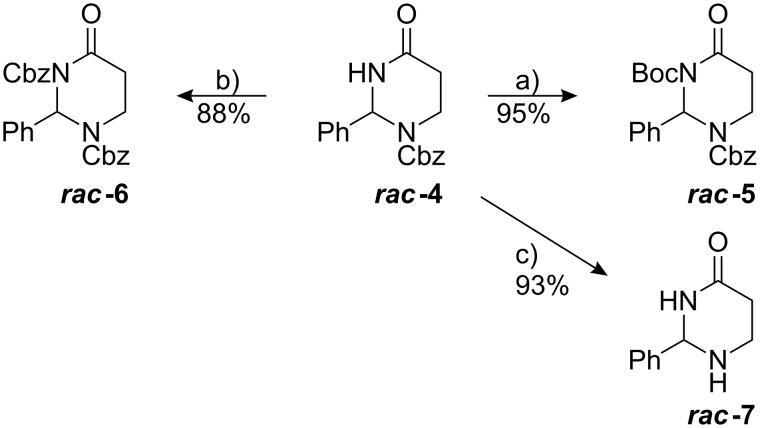
Protection and deprotection of **4**. a) Boc_2_O, DMAP, CH_3_CN, 16 h, rt; b) 1. *n*-BuLi, THF, −78 °C, 30 min; 2. Cbz-Cl, rt, 16 h; c) H_2_, 10% Pd/C, abs. THF, 4 h, rt.

To facilitate our selective ring opening concept, the *N^3^*-nitrogen of **4** had to be protected as a carbamate. While the introduction of a Boc-protecting group was straightforwardly carried out under mild conditions by reaction of **4** with Boc_2_O/DMAP in acetonitrile [41], comparative Cbz-protection required more drastic conditions, i.e. deprotonation of **4** with *n*-BuLi and subsequent reaction with Cbz-Cl (Scheme 3).

In contrast to the reported separation of racemic 2-*tert*-butyl-tetrahydropyrimidine-4(*H*)-ones [37], attempts to resolve 2-phenyl-tetrahydropyrimidine-4(1*H*)-ones ***rac*-4** and ***rac*-5** via HPLC on chiral phases (Chiralcel OD^®^, Chiralpak AD^®^, elution with *i*-PrOH/hexane) were not successful. Interestingly, the *N^1^*-Cbz-protective group of **4** can be chemoselectively cleaved by carefully monitored hydrogenolysis on 10% Pd/C in dry THF solution. The pure fully deprotected tetrahydropyrimidinone ***rac*-7** was obtained after recrystallisation from ethyl acetate. Although **7** proved to be stable under inert gas atmosphere, prolonged contact to air moisture results in slow hydrolysis of the aminal. Attempts to obtain diastereomeric salts of **7** failed. This result corresponds well to the finding that salts of *N^1^*-uncapped tetrahydropyrimidinones are generally unstable [34] – unlike the stable salts of five membered imidazolidinone rings [42].

**Scheme 4:**
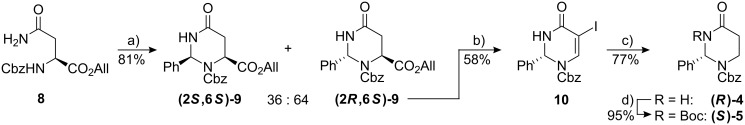
Synthesis of enantiopure 2-phenyl-tetrahydropyrimidine-4(1*H*)-ones. a) PhCH(OMe)_2_, BF_3_·Et_2_O (6.0 equiv), CH_2_Cl_2_, −30 °C → −15 °C, 16 h; b) 1. [Pd(PPh_3_)_4_], morpholine, THF, rt, 1 h; 2. DIB, I_2_, CH_2_Cl_2_, rt, 4 h, then BF_3_·Et_2_O, rt, 1 h; c) Ni(OAc)_2_·4H_2_O/NaBH_4_, MeOH/THF, 0 °C, 10 min; d) Boc_2_O, DMAP, CH_3_CN, 16 h, rt.

Finally, enantiopure 2-phenyltetrahydropyrimidine-4(1*H*)-ones were successfully synthesised via a chiral pool approach by condensing Cbz-L-Asn-OAll (**8**) with benzaldehyde dimethylacetal (Scheme 4). Compared to the respective cyclisation reaction starting from **3**, complete conversion of **8** required a larger excess of BF_3_·Et_2_O, most probably due to the larger number of Lewis basic functions present in the starting material. Starting at −30 °C, the temperature of the reaction mixture had to be raised to −15 °C to enhance the solubility of the starting material as well as to increase the otherwise very low reaction rate. Cyclocondensation afforded the *trans*-configured major product **(2*R*,6*S*)-9** and the *cis*-configured minor diastereomer **(2*S*,6*S*)-9** in a ratio of 64 : 36 (28% *de*). Both compounds were obtained as colourless thick oils after separation by column chromatography. Since the cyclisation reaction was carried out under kinetic control, its moderate *trans*-selectivity contrasts to the high *cis*-selectivity observed for the thermodynamically controlled cyclocondensation of potassium asparaginate and aliphatic aldehydes [25,27–28,31,34–35].

**Figure 1:**
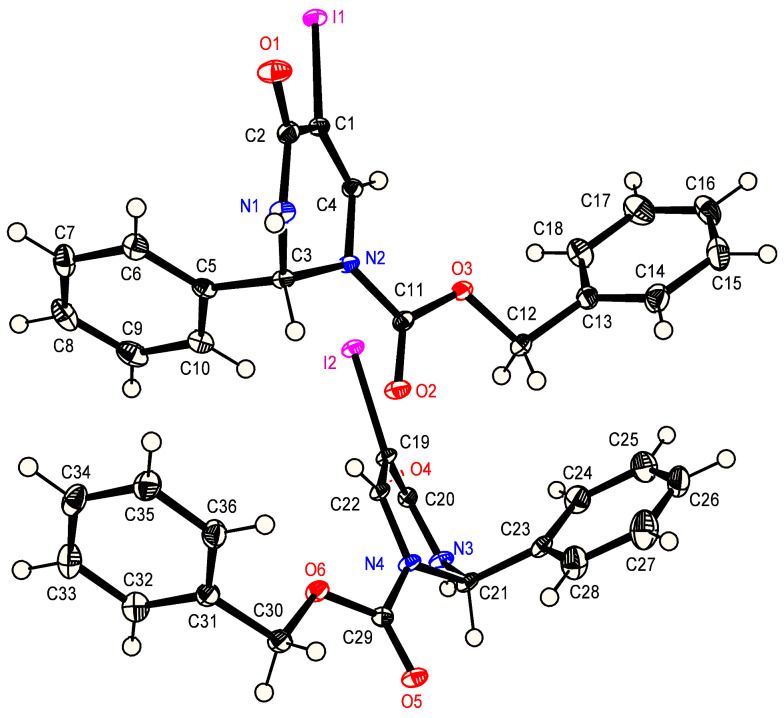
X-ray crystal structure of **10** [44].

En route to the target compound **(*R*)-4**, the allyl ester **(2*R*,6*S*)-9** was cleaved in a Pd(0)-catalysed reaction [43]. In accordance with a literature procedure, the intermediate carboxylic acid was directly subjected to an oxidative decarboxylation [30]. The iodoalkene **10** was obtained by reaction of the free carboxylic acid with diacetoxyiodosobenzene (DIB) in the presence of iodine and BF_3_·Et_2_O. The absolute configuration of the highly crystalline compound **10** was determined in the course of an X-ray analysis [44]. The six-membered heterocycle of **10** shows a flattened boat- to envelope-like conformation. Five of the six ring atoms are almost coplanar while the C^2^-atom and the pseudoaxial C^2^-phenyl group are situated above the plane (Figure 1).

In order to convert the iodinated dihydropyrimidinone **10** into the fully saturated tetrahydropyrimidinone **(*R*)-4**, selective hydrodehalogenation and reduction of the double bond had to be accomplished. Chemoselective reduction of **10** was carried out by reaction with sodium borohydride in the presence of Ni(OAc)_2_. The “nickel boride” formed as black precipitate serves as a highly active hydrogenation catalyst, whereas an excess of NaBH_4_ serves as hydrogen source [45–46]. The reaction reaches completion within ten minutes at 0 °C – virtually without formation of by-products. A similar procedure has formerly been employed by Bella et al. for the reduction of α-halogenated, α,β-unsaturated lactones [47].

**Scheme 5:**
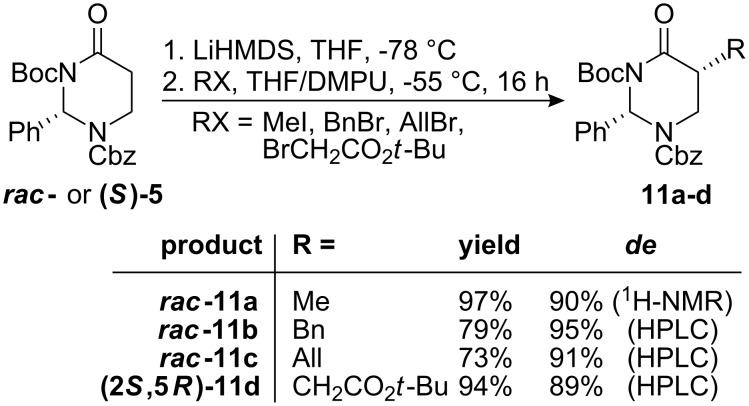
Diastereoselective alkylation of **5**.

The racemic fully protected heterocycle ***rac*-5** as well as the enantiopure compound **(*S*)-5** derived from **(*R*)-4** were both used a starting materials for diastereoselective alkylation experiments. Attempts to enolise **5** at −78 °C by reaction with NaHMDS or LiHMDS, followed by addition of benzyl bromide did not lead to any alkylation product. Instead, the starting material was re-isolated. Addition of sodium iodide to the reaction mixture for an in situ conversion of the alkyl bromide to the alkyl iodide [48] did not increase reactivity. The alkylation reaction started to proceed only after warming the lithium enolate solution to −55 °C, but still remained incomplete after 20 h reaction time. By adding DMPU as co-solvent [49], the alkylation was typically complete within 16 h at −55 °C. The alkylation products **11a-d** were obtained in good yields and with high diastereomeric excesses around 90% (see Scheme 5). However, none of the diastereomeric mixtures was separable by column chromatography on silica gel. Although in general no α,α-dialkylation products were detected by ESI-mass spectrometry, HPLC-analysis of **11d** revealed the existence of a less polar by-product (see Supporting Information File 1), which could probably be explained by additional side chain alkylation of **11d**.

**Figure 2:**
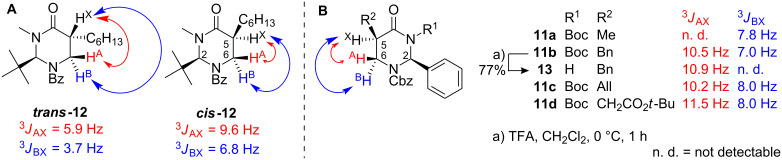
Comparison of coupling constants (C^5^–C^6^-ABX system) within the ^1^H-NMR spectra of literature known compounds ***trans*-12** and ***cis*-12** (**A**) [36] to those of alkylation products **11a–d** and **13** (**B**).

Interestingly, alkylation of **5** turned out to be *syn*-selective, whereas literature known alkylations of the closely related compound **2** selectively afford *anti*-configured alkylation products [25–26,35]. First hints at the stereochemical outcome of the alkylation reaction were deduced by comparing ^1^H NMR spectra of compounds **11a-d** with those of literature known *N^3^*-methylated alkylation products **12** and **14** (Figure 2) [36]. Overall, chemical shifts and coupling constants are quite similar for both types of compounds, indicating an analogous ring conformation in CDCl_3_ solution. In case of **11d**, ^3^*J*-coupling constants within the ABX-system of the three protons at C^5^ and C^6^ parallel those of the *syn*-configured compound ***cis*-12**, but not those of the *anti*-configured compound ***trans*-12** [36]. The high value of the ^3^*J*_AX_-coupling constant of around 10 Hz does not significantly change after removing the *N^3^*-Boc-protective group, as exemplified by compounds **11b/13**.

**Figure 3:**
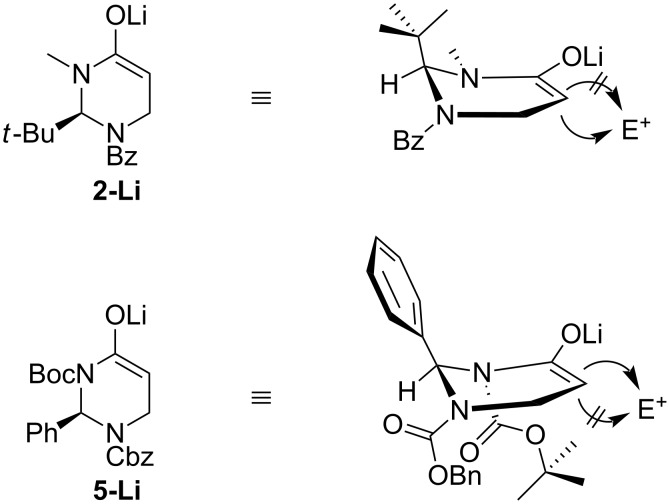
Supposed enolate conformations.

The decisive influence of *N*-acyl substituents within cyclic five- and six-membered *N*,*N*-and *N*,*O*-acetals on the stereochemical course of alkylations and ring closure reactions is a long known and frequently observed phenomenon (for an overview, see ref. [50]). The at first sight surprising differences in stereochemical outcome of alkylations of **2** and **5** could be explained by comparing their proposed enolate conformations (see Figure 3). In case of **2-Li**, the pseudo-axial C^2^-*tert*-butyl function effectively shields one diastereotopic face, thereby enforcing an electrophilic attack at the opposite side of the ring [26]. In contrast to that, the sterically less demanding C^2^-phenyl group within **5-Li** does not completely shield its diastereotopic half room. Instead, the opposite face is more efficiently shielded by the two bulky *N^1^*- and *N^3^*-urethanes. This hypothesis is supported by the fact that alkylation of the *N^3^*-Cbz-protected heterocycle ***rac*-6** resulted in a decreased diastereoselectivity compared to reactions of the *N^3^*-Boc-protected compound **5** (results not shown here). The steric hindrance caused by the C^2^-phenyl ring may nevertheless account for the decrease in reactivity and diastereoselectivity being observed for alkylations of **5** compared to alkylations of **2**.

**Scheme 6:**
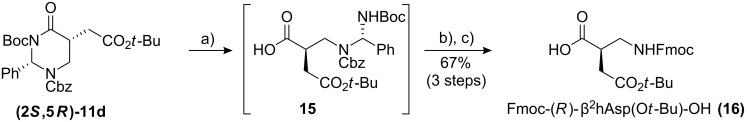
Selective ring opening of the heterocycle **11d** and isolation of orthogonally protected β^2^-homoaspartate **16**. a) LiOH/H_2_O_2_, THF/H_2_O, 0 °C → rt, 5 h, then aq. Na_2_SO_3_, 20 min; b) H_2_, 10% Pd/C, MeOH, rt, 16 h; c) FmocOSu, NaHCO_3_, acetone/H_2_O, rt, 4 h.

To release the *tert*-butyl protected β^2^-homoaspartate, its precursor **11d** was regioselectively degraded in a two step procedure (Scheme 6). Treatment of **11d** with LiOH/H_2_O_2_ in THF/H_2_O afforded the ring opening product **15**. Although **15** can be purified by column chromatography, it turned out to be only moderately stable and slowly degraded to Cbz-(*R*)-β^2^hAsp(O*t*-Bu)-OH, benzaldehyde and *tert*-butyl carbamate upon prolonged exposure to air moisture. Therefore, unpurified **15** was directly converted to the free amino acid by means of hydrogenation. Subsequent Fmoc-protection afforded the orthogonally protected target compound Fmoc-(*R*)-β^2^hAsp(O*t*-Bu)-OH (**16**). The specific rotation of the (*R*)-configured compound **16** in CHCl_3_ showed a value of −1.2, whereas Seebach et al. report a value of +1.4 for the corresponding (*S*)-enantiomer [51]. Thus, the determined optical activity of **16** corresponds well to its expected enantiomeric excess of 89% and doubtlessly confirms the anticipated stereochemistry of the alkylation reaction.

## Conclusion

Within this article, novel procedures are presented to efficiently synthesise 2-phenyl-substituted tetrahydropyrimidine-4(1*H*)-ones. In contrast to literature known 2-alkyl-tetrahydropyrimidine-4(1*H*)-ones, the *N^1^*- and *N^3^*-carbamate functionalised compound **5** is selectively cleavable under mild conditions and can thus be considered as a versatile precursor for functionalised β^2^-amino acids. Starting from L-asparagine, this novel concept was applied to synthesise orthogonally protected (*R*)-β^2^-homoaspartate by means of “self-regeneration of stereogenic centres” (SRS).

Asparagine derived benzaldehyde *N*,*N*-acetals comparable to **9** have formerly been used as α-alkyl-asparagine precursors [39] and furthermore have been employed in peptide chemistry as asparagine protective groups [52] and as proline mimetics [40]. The class of compounds described in this paper can therefore be considered as a versatile tool in peptide and amino acid chemistry.

## Supporting Information

File 1 Detailed synthetic procedures and characterisation data for all new compounds reported in this paper.
