# Supplementary material for: 2-Phenyl-tetrahydropyrimidine-4(1H)-ones – cyclic benzaldehyde aminals as precursors for functionalised β2-amino acids
Source: Beilstein J Org Chem. 2009 Sep 14;5:43. doi: 10.3762/bjoc.5.43 (PMC2779662; doi:10.3762/bjoc.5.43)
Supplement: File 1 — Detailed synthetic procedures and characterisation data for all new compounds reported in this paper. [file Beilstein_J_Org_Chem-05-43-s001.pdf]

Supporting Information

2-Phenyl-tetrahydropyrimidine-4(1*H*)-ones – cyclic benzaldehyde aminals as precursors for functionalised β^2-amino acids

Markus Nahrwold,[1] Arvydas Stončius,[1] Anna Penner,[1] Beate Neumann,[2] Hans-Georg Stammler,[2] and Norbert Sewald [1] *

Bielefeld University, Department of Chemistry, Organic and Bioorganic Chemistry [1] and Inorganic Chemistry [2]

Universitätsstraße 25, D-33615 Bielefeld, Germany

* norbert.sewald@uni-bielefeld.de

General information

All experiments requiring the absence of moisture and/or oxygen were carried out under a static argon atmosphere in flame dried glassware stoppered with rubber septa. Temperatures between – 15 °C and – 55 °C were maintained by a LAUDA RLS-6 cryostat. Dichloromethane and DMPU were dried over calcium hydride, THF was dried by distillation from sodium/benzophenone. Allyl bromide, benzyl bromide and triethylamine were distilled from calcium hydride, *tert*-butyl bromoacetate was distilled from calcium oxide. Benzaldehyde and benzaldehyde dimethyacetal were fractionally distilled prior to use. All other chemicals, reagents and solvents were used as received. MACHEREY NAGEL silica gel "Kieselgel 60" was used as stationary phase for column chromatography (70-230 mesh) and flash chromatography (230-400 mesh). TLC plates (MERCK Kieselgel 60, F_{254} on aluminium foil) were stained either by dipping into a 5 % solution of ninhydrine in ethanol or into ammoniumheptamolybdate/cerium(IV)sulfate reagent [10.0 g $(NH_4)_6Mo_7O_{24} \cdot 4\,H_2O$, 0.4 g $Ce(SO_4)_2$, 5.4 mL conc. H_2SO_4, 180 mL H_2O], followed by subsequent heating, respectively.

NMR-spectra were recorded at 298 K in $CDCl_3$ (standard: TMS) or in DMSO-d_6 (standard: DMSO-CH_3, ^{1}H: 2.50 ppm, ^{13}C: 39.52 ppm [1]) using the BRUKER instruments ARX 250 (^{1}H: 250 MHz, ^{13}C: 63 MHz) and DRX 500 (^{1}H: 500 MHz, ^{13}C: 126 MHz). IR spectra were measured on a JASCO FT/IR-410 instrument. Solids were prepared as KBr disks, liquids as films between NaCl plates. ESI mass spectra were recorded on a BRUKER DALTONIK ESQUIRE 3000 mass spectrometer. High resolution mass spectra (HRMS) were recorded on a BRUKER DALTONIK APEX III FT-ICR-mass spectrometer

equipped with a (nano-)ESI ion source. Melting points were measured in open capillaries on a BÜCHI MELTING POINT B-540 apparatus. All reported values are uncorrected. Specific rotations $[\alpha]_D^{RT}$ $[10^{-1}\ \mathrm{deg\,cm^2\,g^{-1}}]$ were determined at $\lambda = 589$ nm using a JASCO DIP-360 digital polarimeter. Analytical HPLC was performed on a THERMO SEPARATION PRODUCTS HPLC unit (controller SN 4000, pump P 4000, autosampler AS 100, detector UV 6000 LP, UV-absorption measured at λ = 220 nm and 254 nm), equipped with a PHENOMENEX Jupiter C18 column (dimensions 4.6 mm (ID) × 250 mm, grain size 5 μm). The following eluents and methods were used:

Eluent A: $H_2O/CH_3CN/TFA$ 95 : 5: 0.1 v/v/v.

Eluent B: $H_2O/CH_3CN/TFA$ 5 : 95 : 0.1 v/v/v.

Method M1:

flow rate: 1 mL/min

0 – 3 min		100 % A	0 % B
3 – 35 min	→	0 % A	100 % B
35 – 40 min	→	100 % A	0 % B

Method M2:

flow rate: 1 mL/min

0 – 3 min		90 % A	10 % B
3 – 35 min	→	0 % A	100 % B
30 – 40 min		0 % A	100 % B
40 – 45 min	→	90 % A	10 % B

Syntheses

Cbz-$^{\beta}$Ala-OH

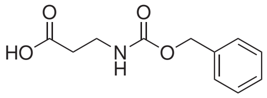

M = 223.23 g mol^{-1} $C_{11}H_{13}NO_4$

An ice cooled solution of β-alanine (26.40 g, 300 mmol) in 1 N aqueous NaOH (300 mL) is treated dropwise with benzyl chloroformate (47.10 mL, 56.30 g, 1.07 equiv.), followed by addition of further 1 N aqueous NaOH (335 mL). The reaction mixture is warmed to rt and stirred overnight. After extracting the basic mixture (pH 10) with Et_2O (3 × 100 mL), the aqueous phase is acidified to pH 2 by addition of concentrated HCl and extracted with CH_2Cl_2 (4 × 200 mL). The combined CH_2Cl_2-extracts are dried over Na_2SO_4, filtered, and concentrated in vacuo. Crystallisation of the residue from EtOAc (100 mL) affords Cbz-$^{\beta}$Ala-OH (59.10 g, 89 %) as colourless crystals. **mp** 104-106 °C (ref. [2] 103-105 °C,

EtOAc/petrol ether). **^{1}H-NMR** (250 MHz, DMSO-d_6): δ [ppm] = 2.40 (t, J = 7.0 Hz, 2H, $C^{\alpha}H_2$), 3.23 (m, 2H, $C^{\beta}H_2$), 5.02 (s, 2H, C$\underline{H_2}$Ph), 7.22-7.40 (m, 6H, C^{ar}H and NH), 12.18 (s, 1H, CO_2H).

Cbz-$^{\beta}$Ala-NH_2 (3)

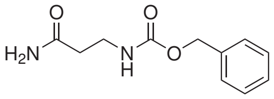

M = 222.24 g mol^{-1} $C_{11}H_{14}N_2O_3$

Ethyl chloroformate (27.63 mL, 31.4 g, 289 mmol) is added at – 15 °C to a solution of Cbz-$^{\beta}$Ala-OH (64.4 g, 288 mmol) and Et_3N (40.2 mL, 29.1 g, 288 mmol) in abs. THF (250 mL) and the reaction mixture is stirred at the same temperature for 20 min. Conc. aq. NH_3 solution (25 %, 80 mL, 3.7 equiv.) is added dropwise and the reaction mixture is left to warm to rt under stirring overnight. The obtained mixture is evaporated to dryness and the residue is recrystallised from EtOH/H_2O (200 mL, 2:1 v/v). After thorough drying in a desiccator over P_2O_5, amide **3** (36.38 g, 57 %) is obtained as a colourless, crystalline solid. **mp** 165-168 °C (ref.[3] 164 °C, EtOH). **^{1}H-NMR** (250 MHz, DMSO-d_6): δ [ppm] = 2.26 (t, J = 7.2 Hz, 2H, $C^{\alpha}H_2$), 3.21 (m, 2H, $C^{\beta}H_2$), 5.02 (s, 2H, C$\underline{H_2}$Ph), 6.80 (br m, 1H, NH), 7.19 (br m, 1H, NH), 7.26-7.40 (m, 6H, C^{ar}H and NH).

rac-1-Benzyl-4-oxo-2-phenyl-dihydropyrimidine-1(2*H*)-carboxylate (*rac*-4)

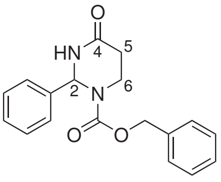

M = 310.35 g mol^{-1} $C_{18}H_{18}N_2O_3$

Method A: Benzaldehyde dimethylacetal (0.40 mL, 0.41 g, 2.70 mmol, 1.2 equiv.) is added to a suspension of **3** (0.50 g, 2.25 mmol) in abs. toluene (50 mL). After addition of a catalytic amount of *p*-TsOH, the reaction mixture is refluxed for 3 h using a Dean-Stark trap. The cold mixture is evaporated to dryness and the residue is taken up in EtOAc (200 mL). The solution is washed with 5 % aq. $KHSO_4$, 10 % aq. $NaHCO_3$, and brine (40 mL each), dried over Na_2SO_4 and evaporated to dryness. Purification by flash chromatography (eluent: EtOAc/hexane 1:1 v/v) affords ***rac*-4** (0.49 g, 73 %) as a light yellow semisolid.

Method B: Benzaldehyde dimethylacetal (0.80 mL, 0.81 g, 5.33 mmol) is added to an ice cooled suspension of **3** (1.00 g, 4.50 mmol) in abs. CH_2Cl_2 (50 mL), followed by dropwise addition of $BF_3 \cdot Et_2O$ (1.13 mL, 1.30 g, 9.19 mmol). A clear yellow reaction solution forms, which is stirred overnight at rt. The reaction is quenched by addition of 5 % aq.

$NaHCO_3$ (20 mL). The reaction mixture decolourises and after gas evolution has ended, the organic phase is washed with 5 % aq. $KHSO_4$, 5 % aq. $NaHCO_3$, and brine (20 mL each), dried over $MgSO_4$, and evaporated to dryness. Crystallisation of the residual yellow oil from EtOAc/petrol ether (15 mL, 1:2 v/v) affords ***rac*-4** (1.20 g, 86 %) as colourless, crystalline solid.

mp 96-98 °C. ^{1}**H-NMR** (250 MHz, $CDCl_3$, TMS): δ [ppm] = 2.31 (ddd, J = 17.3, 5.2, 2.9 Hz, 1H, $C^5\underline{H}^AH^B$), 2.52 (ddd, J = 17.4, 10.9, 7.0 Hz, 1H, $C^5H^A\underline{H}^B$), 3.08 (ddd, J = 13.8, 11.1, 4.9 Hz, 1H, $C^6\underline{H}^AH^B$), 4.02 (m, 1H, $C^6H^A\underline{H}^B$), 5.18 (d, J = 12.1 Hz, 1H, $C\underline{H}^AH^BPh$), 5.26 (d, J = 12.2 Hz, 1H, $CH^A\underline{H}^BPh$), 6.66 (br m, 1H, C^2H), 7.27-7.41 (m, 10H, $C^{ar}H$), 8.06 (br m, 1H, NH). ^{13}**C{^{1}H}-NMR** (63 MHz, $CDCl_3$, TMS): δ [ppm] = 31.3 (C^5H_2), 36.0 (C^6H_2), 65.4 (C^2H), 68.1 ($\underline{C}H_2Ph$), 126.2, 128.1, 128.4, 128.6, 128.8 ($C^{ar}H$), 135.9, 139.1 (C^{ar}) 154.5 ($\underline{C}O_2Bn$), 170.7 (C^4). **IR** (disk, KBr): $\tilde{\nu}$ [cm^{-1}] = 3208 m, 3056 w, 3035 w, 2965 w, 1718 s, 1671 vs, 1493 w, 1474 m, 1453 s, 1424 s, 1397 w, 1351 w, 1334 w, 1313 w, 1273 s, 1214 w, 1193 w, 1179 m, 1104 s, 1069 w, 1035 w, 992 m, 974 m, 933 w, 910 w, 851 w, 812 w, 765 w, 752 m, 740 m, 696 s. **HRMS** (ESI-FT-ICR): *m/z* = 333.1205 (calculated for $[C_{18}H_{18}N_2O_3+Na]^+$: 333.1215); 643.2517 (calculated for $[(C_{18}H_{18}N_2O_3)_2+Na]^+$: 643.2500).

rac-1-Benzyl-3-*tert*-butyl-4-oxo-2-phenyl-dihydropyrimidine-1,3(2*H*,4*H*)-dicarboxylate (*rac*-5)

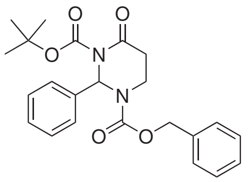

M = 410.46 g mol^{-1} $C_{23}H_{26}N_2O_5$

A solution of ***rac*-4** (1.02 g, 3.29 mmol) and 4-DMAP (40 mg, 0.33 mmol, 0.1 equiv.) in abs. CH_3CN (30 mL) is treated with a solution of Boc_2O (0.86 g, 3.94 mmol, 1.2 equiv.) in abs. CH_3CN (9 mL). The reaction solution is stirred at rt overnight. The solvent is removed in vacuo, the residue is taken up in EtOAc (75 mL), washed with 5 % aq. $KHSO_4$, 5 % aq. $NaHCO_3$, and brine (20 mL each), dried over $MgSO_4$ and evaporated to dryness. The obtained residue is purified by flash chromatography (eluent: EtOAc/petrol ether 1:3 v/v + 0.5 vol% AcOH). ***rac*-5** (1.28 g, 95 %) is obtained as a slightly yellow, highly viscous oil. **HPLC**: t_R = 31.3 min (method M1). ^{1}**H-NMR** (250 MHz, $CDCl_3$, TMS): δ [ppm] = 1.49 (s, 9H, $C(CH_3)_3$), 2.36 (m, 1H, $C^5\underline{H}^AH^B$), 2.55 (br m, 1H, $C^5H^A\underline{H}^B$), 3.43 (m, 1H, $C^6\underline{H}^AH^B$), 3.89 (br m, 1H, $C^6H^A\underline{H}^B$), 5.23 (d, J = 12.3 Hz, 1H, $C\underline{H}^AH^B$-Ph), 5.29 (d, J = 12.3 Hz, 1H, $CH^A\underline{H}^BPh$), 7.14-7.41 (m, 10H, $C^{ar}H$), 7.53 (br m, 1H,

C^2H). ^{13}**C{^{1}H}-NMR** (63 MHz, $CDCl_3$, TMS): δ [ppm] = 27.9/28.0 (C($\underline{C}H_3)_3$), 33.1/38.4 (C^5H_2), 38.2 (C^6H_2), 67.7 (C^2H), 68.1 ($\underline{C}H_2Ph$), 84.2 ($\underline{C}(CH_3)_3$), 125.3, 128.0 (2 overlapping sigals), 128.4, 128.6, 129.1 ($C^{ar}H$), 135.9, 138.3 (C^{ar}), 150.5, 154.4/155.3 ($\underline{C}O_2Bn$), 168.6 (C^4). **IR** (film, NaCl): $\tilde{\nu}$ [cm^{-1}] = 3089 m, 3063 m, 3032 m, 2980 s, 1778 s, 1718 vs, 1602 w, 1586 w, 1496 m, 1452 m, 1410 s, 1369 s, 1292 s, 1149 s, 1117 m, 1080 w, 1053 w, 1031 w, 1001 m, 911 w, 858 m, 808 w, 791 w, 767 m, 735 m, 698 s. **MS** (ESI, cation mode): *m/z* = 433.0 (calculated for $[(C_{23}H_{26}N_2O_5+Na]^+$: 433.2), 843.1 (calculated for $[(C_{23}H_{26}N_2O_5)_2+Na]^+$: 843.4). **HRMS** (ESI-FT-ICR): *m/z* = 465.1992 (calculated for $(C_{23}H_{26}N_2O_5 + CH_3OH + Na]^+$: 465.1983); 907.4101 (calculated for $[(C_{23}H_{26}N_2O_5 + CH_3OH)_2 + Na]^+$: 907.4127).

rac-Dibenzyl-6-oxo-2-phenyl-dihydropyrimidine-1,3(2*H*,4*H*)-dicarboxylate (*rac*-6)

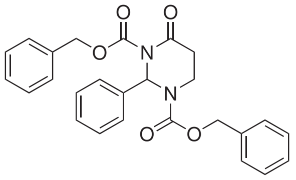

M = 444.48 g mol^{-1} $C_{26}H_{24}N_2O_5$

n-BuLi (1.6 M solution in hexane, 2.20 mL, 3.52 mmol, 1.1 equiv.) is added dropwise at – 78 °C to a solution of ***rac*-4** (1.00 g, 3.22 mmol) in abs. THF (20 mL). After stirring the reaction mixture for 30 min, a solution of benzyl chloroformate (0.50 mL, 0.61 g, 3.55 mmol, 1.1 equiv.) in abs. THF (8 mL) is added dropwise. The reaction mixture is left to reach room temperature under stirring overnight. The reaction is quenched by addition of saturated NH_4Cl-solution (30 mL), the phases are separated and the aqueous phase is extracted with EtOAc (3 × 30 mL). The combined organic phases are washed with brine, dried over $MgSO_4$ and evaporated to dryness. Purification of the residue by flash chromatography (eluent: EtOAc/hexane 1:3 v/v + 0.5 vol % HOAc) affords ***rac*-6** (1.26 g, 88 %) as a colourless, highly viscous oil. ^{1}**H-NMR** (250 MHz, $CDCl_3$, TMS): δ [ppm] = 2.39 (ddd, J = 16.9, 8.9, 7.9 Hz, 1H, $C^5\underline{H}^AH^B$), 2.55 (br m, 1H, $C^5H^A\underline{H}^B$), 3.44 (m, br, 1H, $C^6\underline{H}^AH^B$), 3.89 (m, br, 1H, $C^6H^A\underline{H}^B$), 5.08-5.28 (m, 4H, 2 × $C\underline{H}_2Ph$), 7.15-7.42 (m, 15 H, $C^{ar}H$), 7.64 (br m, 1H, C^2H). ^{13}**C{^{1}H}-NMR** (63 MHz, $CDCl_3$, TMS): δ [ppm] = 33.1 (C^5H_2), 38.2 (C^6H_2), 68.0 (C^2H), 68.2, 69.0 ($\underline{C}H_2Ph$), 125.3, 128.0, 128.4, 128.6, 129.1 ($C^{ar}H$), 135.0, 135.8, 137.9 (C^{ar}), 152.3, 154.4/155.2 ($\underline{C}O_2N$), 168.4 (C^4). **IR** (Film, NaCl): $\tilde{\nu}$ [cm^{-1}] = 3063 w, 3032 w, 2957 w, 1783 s, 1722 vs, 1586 w, 1497 m, 1453 m, 1411 s, 1376 s, 1281 s, 1240 s, 1157 w, 1117 m, 1064 w, 1030 w, 1002 m, 915 w, 851 w, 800 w, 738 m, 698 s. **MS** (ESI, cation mode): *m/z* = 445.1 $[M+H]^+$ (calculated for $[C_{26}H_{25}N_2O_5]^+$: 445.2).

rac-2-Phenyltetrahydropyrimidine-4(1*H*)-one (*rac*-7)

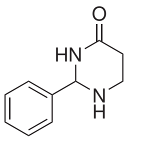

M = 176.22 g mol^{-1} $C_{10}H_{12}N_2O$

Pd/C-catalyst (10 % Pd, 0.10 g) is added to a solution of ***rac*-4** (1.00 g, 3.22 mmol) in abs. THF (50 mL) and the resulting mixture is stirred for 3 h under an H_2-atmosphere (balloon, 1 bar). The hydrogen is removed by purging with argon, the reaction mixture is filtered through a small plug of Celite®, and the clear solution is evaporated to dryness. Recrystallisation of the residue from EtOAc/hexane (40 mL, 3:1 v/v) yields ***rac*-7** (0.53 g, 93 %) as colourless crystals. **mp** 134-135 °C. ^{1}**H-NMR** (500 MHz, DMSO-d_6): δ [ppm] = 2.12 (ddd, *J* = 17.1, 4.8, 4.8 Hz, 1H, $C^5\underline{H}^AH^B$), 2.21 (ddd, *J* = 17.1, 8.2, 6.5 Hz, 1H, $C^5H^A\underline{H}^B$), 2.76-2.92 (m, 3H, $C^6\underline{H_2}N\underline{H}$), 5.18 (br m, 1H, C^2H), 7.29-7.41 (m, 5H, $C^{ar}H$), 8.01 (s, 1H, C(O)NH). ^{13}**C{^{1}H}-NMR** (126 MHz, DMSO-d_6): δ [ppm] = 32.3 (C^5H_2), 39.8 (C^6H_2), 69.7 (C^2H), 126.9, 127.8, 128.1 ($C^{ar}H$), 141.8 (C^{ar}), 169.6 (C=O). **IR** (disk, KBr): $\tilde{\nu}$ [cm^{-1}] = 3466 w, 3291 s, 3152 m, 2949 w, 2883 w, 2810 w, 1649 vs, 1604 vs, 1475 s, 1455 w, 1408 m, 1346 m, 1325 m, 1298 m, 1284 m, 1239 m, 1204 w, 1190 w, 1125 m, 1085 w, 1046 m, 1006 m, 929 w, 914 w, 842 s, 806 m, 759 m, 709 m. **HRMS** (ESI-FT-ICR): *m/z* = 199.0840 (calculated for $[C_{10}H_{12}N_2O+Na]^+$: 199.0842); 375.1785 (calculated for $[(C_{10}H_{12}N_2O)_2+Na]^+$: *m/z* = 375.1797).

(*S*)-2-Benzyloxycarbonylaminosuccinic acid 1-allylester-4-amide (8)

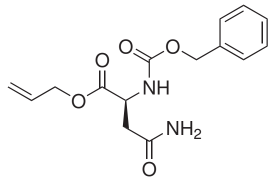

M = 306.31 g mol^{-1} $C_{15}H_{18}N_2O_5$

A vacuum dried mixture of *N*-Cbz-L-asparagine (5.00 g, 18.78 mmol) and $NaHCO_3$ (3.16 g, 37.56 mmol, 2 equiv.) is taken up in abs. DMF (30 mL). Allyl bromide (2.43 g, 1.70 mL, 20.09 mmol) is added and the reaction mixture is stirred for 48 h at rt. The solvent is removed in vacuo and the residue is partitioned between H_2O and EtOAc (50 mL each). The aqueous phase is extracted with EtOAc (2 × 50 mL), the combined organic phases are washed with saturated aq. $NaHCO_3$ and brine (25 mL each), dried over $MgSO_4$ and evaporated to dryness. Recrystallisation from EtOAc/cyclohexane (40 mL, 1:1 v/v) affords **8** (3.86 g, 68 %) as colourless crystals. **mp** 130-131 °C. $[\alpha]_D^{RT}$ = +9.7 (c = 1.0 in $CHCl_3$). ^{1}**H-NMR** (250 MHz, DMSO-d_6): δ [ppm] = 2.50 (dd, *J* = 15.1, 7.6 Hz, 1H,

$C^{\beta}\underline{H}^{A}H^{B}$), 2.61 (dd, J = 15.5, 5.7 Hz, 1H, $C^{\beta}H^{A}\underline{H}^{B}$), 4.48 (ddd, J = 7.8, 7.6, 6.0 Hz, 1H, $C^{\alpha}\underline{H}$), 4.54-4.60 (m, 2H, $C\underline{H}_2CH{=}CH_2$), 5.04 (s, 2H, $C\underline{H}_2Ph$), 5.19 (dm, J = 10.5 Hz, 1H, $CH{=}C\underline{H}_{2(trans)}$), 5.30 (dm, J = 17.3 Hz, 1H, $CH{=}C\underline{H}_{2(cis)}$), 5.88 (dddd, J = 17.1, 10.5, 5.3, 5.2 Hz, 1H, $CH_2{=}C\underline{H}$), 6.93 (s, 1H, NH), 7.31-7.40 (m, 6H, $C^{ar}H$ and NH), 7.62 (d, J = 8.2 Hz, 1H, NH). **^{13}C{^{1}H}-NMR** (63 MHz, DMSO-d_6): δ [ppm] = 36.6 ($\underline{C}^{\beta}H_2$), 50.7 ($C^{\alpha}H$), 64.9 ($\underline{C}H_2CH{=}CH_2$), 65.5 ($\underline{C}H_2Ph$), 117.4 ($CH{=}\underline{C}H_2$), 127.6, 127.8, 128.3 ($C^{ar}H$), 132.3 ($\underline{C}H{=}CH_2$), 136.8 ($C^{ar}$), 155.8 (C(O)NH), 170.8 ($C(O)NH_2$), 171.3 ($\underline{C}O_2All$). **IR** (disk, KBr): $\tilde{\nu}$ [cm^{-1}] = 3421 s, 3344 s, 3309 s, 3208 m, 3064 w, 2956 w, 1757 s, 1740 s, 1699 vs, 1683 s, 1663 vs, 1542 s, 1419 m, 1365 w, 1336 m, 1315 w, 1286 s, 1254 m, 1218 s, 1204 s, 1173 m, 1121 w, 1066 s, 990 m, 959 w, 935 m, 842 w, 783 w, 757 w, 733 m, 697 m, 649 m.

(2*R*,6*S*)- and (2*S*,6*S*)-4-Oxo-2-phenyltetrahydropyrimidine-1,6(2*H*)-dicarboxylic acid 6-allyl ester 1-benzyl ester (9)

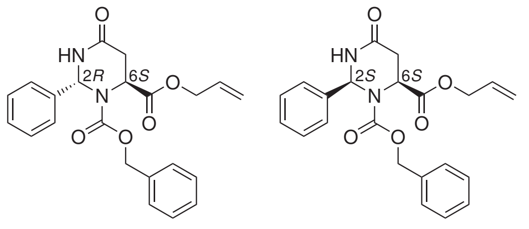

M = 394.42 g mol^{-1} $C_{22}H_{22}N_2O_5$

A solution of **8** (4.00 g, 13.04 mmol) and benzaldehyde dimethylacetal (2.04 mL, 13.64 mmol) in abs. CH_2Cl_2 (120 mL) is cooled to – 30 °C. $BF_3 \cdot Et_2O$ (11.12 g, 9.84 mL, 78.4 mmol, 6 equiv.) is added dropwise and the reaction mixture is stirred for 16 h at – 15 °C. The mixture is poured into saturated aq. $NaHCO_3$ (300 mL) and stirred until gas evolution ends. The aqueous phase is extracted with CH_2Cl_2 (2 × 300 mL), the combined organic phases are washed with 5 % aq. $KHSO_4$, saturated aq. $NaHCO_3$, and brine (100 mL each), dried over $MgSO_4$ and evaporated to dryness. The diastereomeric mixture is resolved by flash chromatography (eluent EtOAc/petrol ether 1:2 → 1:1 v/v). **(2*R*,6*S*)-9** (1st fraction, 2.69 g, 52 %) and **(2*S*,6*S*)-9** (2nd fraction, 1.50 g, 29 %) are obtained as highly viscous, colourless oils.

Physical data of **(2*R*,6*S*)-9**: $[\alpha]_D^{RT}$ = + 12.1 (c = 1.10 in $CHCl_3$). **HPLC**: t_R = 27.4 min (method M2). **^{1}H-NMR** (250 MHz, DMSO-d_6): δ [ppm] = 2.38 (dm, J = 16.4 Hz, 1H, $C^{5}\underline{H}^{A}H^{B}$), 2.60 (dd, J = 16.4, 7.3 Hz, 1H, $C^{5}H^{A}\underline{H}^{B}$), 4.44-4.68 (br m, 2H, $C\underline{H}_2CH{=}CH_2$), 4.96 (br m, 1H, $C^{6}H$), 5.11 (d, J = 12.6 Hz, 1H, $C\underline{H}^{A}H^{B}Ph$), 5.16 (dm, J = 11.1 Hz, 1H, $CH{=}C\underline{H}^{A}H^{B}$), 5.17 (d, J = 12.8 Hz, 1H, $CH^{A}\underline{H}^{B}Ph$), 5.25 (dm, J = 17.6 Hz, 1H, $CH{=}CH^{A}\underline{H}^{B}$), 5.79 (br m, 1H, $CH_2{=}C\underline{H}$), 6.26 (d, J = 5.4 Hz, 1H, $C^{2}H$), 7.19-7.43 (m, 10H, $C^{ar}H$), 9.06 (br m, 1H, NH). **^{13}C{^{1}H}-NMR** (63 MHz, DMSO-d_6): δ [ppm] = 33.7

(C^5H_2), 53.7 (C^6H), 64.9 (C^2H), 65.3 ($\underline{C}H_2CH{=}CH_2$), 67.2 ($\underline{C}H_2Ph$), 117.8 ($CH{=}\underline{C}H_2$), 125.4, 127.5, 127.9, 128.3, 128.6 ($C^{ar}H$), 131.9 ($\underline{C}H{=}CH_2$), 136.0, 141.3 (C^{ar}), 154.2 ($\underline{C}O_2Bn$), 167.3 (C^4), 170.5 ($\underline{C}O_2All$). **IR** (film, NaCl): $\tilde{\nu}$ [cm^{-1}] = 3537 vs, 3316 br m, 3064 w, 3032 w, 2950 w, 1695 vs, 1586 w, 1496 m, 1454 s, 1409 s, 1336 s, 1184 s, 1130 w, 1072 m, 1016 m, 934 s, 844 w, 794 w, 739 m, 699 s, 610 m. **HRMS** (ESI-FT-ICR): *m/z* = 417.1418 (calculated for $[C_{22}H_{22}N_2O_5{+}Na]^+$: 417.1408).

Physical data of **(2*S*,6*S*)-9**: $[\alpha]_D^{RT}$ = −23.5 (c = 1.00 in $CHCl_3$). **HPLC**: t_R = 27.1 min (method M2). **^{1}H-NMR** (250 MHz, DMSO-d_6): δ [ppm] = 2.25 (dd, *J* = 15.7, 10.8 Hz, 1H, $C^5\underline{H}^AH^B$), 2.49 (dd, *J* = 15.8, 6.0 Hz, 1H, $C^5H^A\underline{H}^B$), 4.18-4.48 (br m, 2H, $C\underline{H_2}CH{=}CH_2$), 4.82 (dd, *J* = 10.8, 6.0 Hz, 1H, C^6H), 5.12-5.25 (m, 4H, $C\underline{H_2}Ph$ and $C\underline{H_2}{=}CH$), 5.70 (br m, 1H, $CH_2{=}C\underline{H}$), 6.41 (br m, 1H, C^2H), 7.28-7.49 (m, 10H, $C^{ar}H$), 9.04 (br m, 1H, NH). **^{13}C{^{1}H}-NMR** (63 MHz, DMSO-d_6): δ [ppm] = 32.6 (C^5H_2), 53.4 (C^6H), 64.5 (C^2H), 65.2 ($\underline{C}H_2CH{=}CH_2$), 67.5 ($\underline{C}H_2Ph$), 118.1 ($CH{=}\underline{C}H_2$), 126.0, 127.6, 128.0, 128.28, 128.35 ($C^{ar}H$), 131.8 ($\underline{C}H{=}CH_2$), 136.0, 140.0 (C^{ar}), 154.5 ($\underline{C}O_2Bn$), 167.8 (C^4), 169.8 ($\underline{C}O_2All$). **IR** (film, NaCl): $\tilde{\nu}$ [cm^{-1}] = 3537 vs, 3290 br m, 3064 w, 2946 w, 1709 vs, 1497 m, 1453 m, 1413 m, 1308 s, 1182 s, 1088 m, 1013 w, 932 s, 878 w, 820 w, 795 w, 742 m, 698 s. **MS** (ESI, cation mode): *m/z* = 395.2 $[M{+}H]^+$ (calculated for $[C_{22}H_{23}N_2O_5]^+$: 395.2); 417.2 $[M{+}Na]^+$ (calculated for $[C_{22}H_{22}N_2O_5{+}Na]^+$: 417.1).

(*R*)-5-Iodo-4-oxo-2-phenyl-3,4-dihydro-2*H*-pyrimidine-1(2*H*)-carboxylic acid benzyl ester (10)

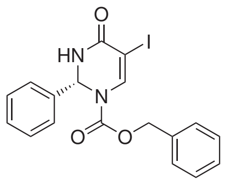

M = 434.23 g mol^{-1} $C_{18}H_{15}IN_2O_3$

Morpholine (0.14 mL, 1.61 mmol, 2.0 equiv.) is added to a solution of allyl ester **(2*R*,6*S*)-9** (317 mg, 0.80 mmol) in abs. THF (8 mL), followed by a solution of $Pd(PPh_3)_4$ (93 mg, 0.08 mmol, 10 mol%) in abs. THF (2 mL). The reaction mixture is stirred for 1 h at rt and subsequently evaporated to dryness. The residue is taken up in saturated aq. $NaHCO_3$ (50 mL) and extracted with EtOAc (3 × 20 mL). The aqueous phase is acidified by addition of 5 % aq. $KHSO_4$ and extracted with EtOAc (1 × 100 mL and 2 × 50 mL). The combined organic extracts of the acidic solution are dried over $MgSO_4$ and evaporated to dryness. High vacuum drying affords the crude carboxylic acid as a white solid foam, which is directly subjected to the following reaction.

In accordance to a literature procedure, [4] a mixture of the freshly prepared carboxylic acid (285 mg, ca. 0.80 mmol), DIB (518 mg, 1.61 mmol, 2.0 equiv.), and iodine (204 mg,

0.80 mmol, 1.0 equiv.) is taken up in abs. CH_2Cl_2 (16 mL). The dark red suspension is stirred for 4 h at rt. After adding $BF_3 \cdot Et_2O$ (0.20 mL, 0.23 g, 1.63 mmol, 2.0 equiv.) the reaction mixture is stirred for 1 h at rt and subsequently diluted with CH_2Cl_2 (25 mL). The mixture is washed with 5 % aq. $Na_2S_2O_3$ (2 × 25 mL), 5 % aq. $NaHCO_3$ (1 × 25 mL), and brine (1 × 25 mL), dried over $MgSO_4$ and evaporated to dryness. Purification of the crude product by column chromatography (eluent: EtOAc/hexane 1:1 v/v) yields **10** (203 mg, 58 % over 2 steps) as light yellow solid. A single recrystallisation from EtOAc affords colourless crystals suitable for X-ray analysis. **mp** 196-197 °C. $[\alpha]_D^{RT}$ = – 183.5 (c = 1.02 in $CHCl_3$). ^{1}**H-NMR** (500 MHz, DMSO-d_6): δ [ppm] = 5.27 (d, J = 12.0 Hz, 1H, C$\underline{H}^A$H^{B}Ph), 5.32 (d, J = 12.2 Hz, 1H, CH$^A\underline{H}^B$Ph), 6.47 (d, J = 4.5 Hz, 1H, C^2H), 7.25-7.45 (m, 10H, $C^{ar}H$), 7.92 (s, 1H, C^6H), 8.99 (d, J = 4.8 Hz, 1H, NH). ^{13}**C{^{1}H}-NMR** (126 MHz, DMSO-d_6): δ [ppm] = 65.6 (C^2H), 68.7 ($\underline{C}H_2Ph$), 74.4 (C^5), 125.5, 128.2, 128.49, 128.55, 128.66, 128.72 ($C^{ar}H$), 135.3, 139.1 (C^{ar}), 140.7 (C^6H), 150.5 (CO_2Bn), 159.5 (C^4). **HRMS** (ESI-FT-ICR): *m/z* = 457.0031 (calculated for $[C_{18}H_{15}IN_2O_3+Na]^+$: 457.0019).

(R)-1-Benzyl-4-oxo-2-phenyl-dihydropyrimidine-1(2H)-carboxylate ((R)-4)

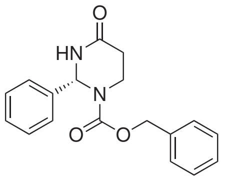

M = 310.35 g mol^{-1} $C_{18}H_{18}N_2O_3$

A mixture of **10** (113 mg, 0.26 mmol) and $Ni(OAc)_2 \cdot 4\,H_2O$ (193 mg, 0.78 mmol, 3.0 eq.) is taken up in abs. THF and abs. MeOH (5 mL, 1 : 1 v/v). The mixture is cooled in an ice bath and $NaBH_4$ (89 mg, 2.35 mmol, 9 equiv.) is added portionwise within 1 min. A black solid precipitates with vigorous gas evolution. The heterogenous mixture is stirred for 10 min in a stoppered flask equipped with a balloon securing pressure balance. After addition of EtOAc (50 mL), the mixture is filtered through a plug of Celite® and the residual solid is washed portionwise with EtOAc (100 mL). The combined filtrates are evaporated to dryness and the residue is purified by column chromatography (eluent: EtOAc/hexane 1:1 v/v → 2:1 v/v + 0.5 vol.-% AcOH). **(*R*)-4** (62 mg, 77 %) is obtained as a colourless, highly viscous oil after coevaporation with toluene and drying in vacuo. $[\alpha]_D^{RT}$ = – 110.0 (c = 0.84 in $CHCl_3$). The spectroscopic data of **(*R*)-4** correspond to those of the racemic material.

(S)-1-Benzyl-3-*tert*-butyl-4-oxo-2-phenyl-dihydropyrimidine-1,3(2H,4H)-dicarboxylate ((S)-5)

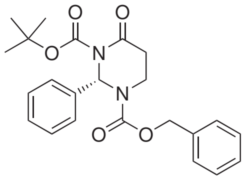

M = 410.46 g mol^{-1} $C_{23}H_{26}N_2O_5$

The synthesis of **(*S*)-5** starts from **(*R*)-4** and corresponds to those of the racemate ***rac*-5** (see p. 4). $[\alpha]_D^{RT} = -1.2$ (c = 1.20 in $CHCl_3$).

Diastereoselective alkylation

General Procedure (GP1)

A solution of **5** (0.25 g, 0.61 mmol) in abs. THF (10 mL) is cooled to – 78 °C. LiHMDS solution (1.06 M in THF, 0.63 mL, 0.67 mmol, 1.1 equiv) is added dropwise and the reaction solution is stirred for 0.5 h at – 78 °C. A solution of the corresponding alkyl halogenide (0.67 mmol, 1.1 equiv) in THF (4 mL) and DMPU (1 mL) is added dropwise. The reaction mixture is stirred for 1 h at – 78 °C and for 16 h at – 55 °C. The reaction is quenched by addition of saturated aq. NH_4Cl (10 ml) and the mixture is warmed to rt under stirring. H_2O (10 mL) is added and the aqueous mixture is extracted with EtOAc (3 × 15 mL). The combined organic extracts are washed with 5 % aq. $KHSO_4$, 5 % aq. $NaHCO_3$, and brine (15 mL each), dried over $MgSO_4$ and evaporated to dryness. Purification by flash chromatography (eluent: EtOAc/hexane 1:3 v/v) affords the corresponding alkylation product **11**.

rac-(*cis*)-1-Benzyl-3-*tert*-butyl-5-methyl-4-oxo-2-phenyl-dihydropyrimidine-1,3(2*H*,4*H*)-dicarboxylate (*rac*-11a)

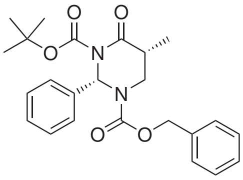

M = 424.49 g mol^{-1} $C_{24}H_{28}N_2O_5$

Reaction of ***rac*-5** and iodomethane according to GP1 affords ***rac*-11a** (0.25 g, 97 %) as a highly viscous colourless oil. *dr* = 95.0 : 5.0 (^{1}H-NMR). **HPLC**: t_R = 33.7 min (method M1). **^{1}H-NMR** (250 MHz, $CDCl_3$, TMS): δ [ppm] = 1.06 (d, J = 6.8 Hz, 3H, $C^5HC\underline{H}_3$), 1.55 (s, 9H, $C(CH_3)_3$), 2.41 (m, 1H, C^5H), 3.48 (m, $C^6\underline{H}^AH^B$), 3.66 (dd, J = 12.0, 7.8 Hz, 1H, $C^6H^A\underline{H}^B$), 5.23 (d, J = 12.4 Hz, 1H, $C\underline{H}^AH^BPh$), 5.29 (d, J = 12.2 Hz, 1H, $CH^A\underline{H}^BPh$), 7.18-7.43 (m, 10H, $C^{ar}H$), 7.61 (br s, 1H, C^2H). **^{13}C{^{1}H}-NMR** (63 MHz, $CDCl_3$, TMS): δ [ppm] = 13.0 ($C^5H\underline{C}H_3$), 28.0 ($C(\underline{C}H_3)_3$), 36.1 (C^5), 46.1/46.4 (C^6), 67.3/67.4 (C^2), 68.0/68.1 ($\underline{C}H_2Ph$), 84.2 ($\underline{C}(CH_3)_3$), 124.9, 128.0, 128.3, 128.6, 129.2 ($C^{ar}H$), 136.0, 138.5/138.6 (C^{ar}), 150.9/151.1, 154.4/155.4 (NCO_2), 171.2/171.3 (C^4). **IR** (film, NaCl): $\tilde{\nu}$ [cm^{-1}] = 3089 w, 3063 w, 3032 m, 2980 s, 2936 m, 2890 w, 1780 s, 1716 vs, 1602 w, 1586 w, 1496 m, 1450 s, 1410 s, 1369 s, 1283 s, 1251 s, 1148 s, 1096 m, 1031 w, 985 m, 965 m, 924 w, 852 m, 769 m, 737 m, 698 s. **HRMS** (ESI-FT-ICR): *m/z* = 447.1889 (calculated for $[C_{24}H_{28}N_2O_5+Na]^+$: 447.1877).

rac-(*cis*)-1,5-Dibenzyl-3-*tert*-butyl-4-oxo-2-phenyl-dihydropyrimidine-1,3(2*H*,4*H*)-dicarboxylate (*rac*-11b)

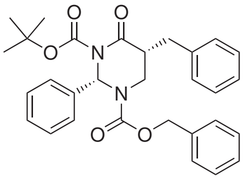

M = 500.59 g mol^{-1} $C_{30}H_{32}N_2O_5$

Reaction of ***rac*-5** and benzyl bromide according to GP1 affords ***rac*-11b** (0.24 g, 79 %) as a highly viscous colourless oil. *dr* = 97.6 : 2.4 (HPLC). **HPLC** (method M1): t_R = 36.9 min (major diastereomer), 37.3 min (minor diastereomer). **^{1}H-NMR** (500 MHz, $CDCl_3$, TMS): δ [ppm] = 1.42/1.53 (2 s, 9H, $C(CH_3)_3$), 2.56 (dd, J = 14.0, 9.5 Hz, 1H, $C^5HC\underline{H}^AH^BPh$), 2.65 (m, 1H, C^5H), 3.24 (dd, J = 14.1, 3.3 Hz, 1H, $C^5HCH^A\underline{H}^BPh$), 3.43 (dd, J = 12.2, 7.0 Hz, 1H, $C^6\underline{H}^AH^B$), 3.55 (dd, J = 11.9, 10.5 Hz, 1H, $C^6H^A\underline{H}^B$), 5.19-5.27 (m, 2H, $OC\underline{H}_2Ph$), 6.96-7.36 (m, 15H, $C^{ar}H$), 7.57 (s, 1H, C^2H). **^{13}C{^{1}H}-NMR** (126 MHz, $CDCl_3$, TMS): δ [ppm] = 26.9/27.9 ($C(\underline{C}H_3)_3$), 34.4/34.6 ($C^5H\underline{C}H_2Ph$),

42.8/43.0 (C^5H), 43.7 (C^6H_2), 67.2/67.4 (C^2H), 67.9/68.1 ($O\underline{C}H_2Ph$), 84.3 ($\underline{C}(CH_3)_3$), 124.9/125.1, 126.5, 127.9, 128.1, 128.3, 128.4, 128.5, 128.6, 128.7, 129.06/129.12 ($C^{ar}H$), 135.7, 137.9/138.0, 138.3/138.4 (C^{ar}), 150.6/151.0, 154.3/155.3 (NCO_2), 170.5/170.7 (C^4). **IR** (film, NaCl): $\tilde{\nu}$ [cm^{-1}] = 3063 w, 3031 m, 2980 m, 1778 s, 1719 vs, 1603 w, 1585 w, 1496 m, 1453 m, 1409 m, 1369 m, 1291 m, 1147 s, 1120 m, 1080 w, 1030 w, 1003 w, 956 m, 916 m, 852 m, 809 w, 735 s, 698 s. **HRMS** (ESI-FT-ICR): *m/z* = 523.2204 (calculated for $[C_{30}H_{32}N_2O_5+Na]^+$: 523.2190); 1023.4522 (calculated for $[(C_{30}H_{32}N_2O_5)_2+Na]^+$: 1023.4552).

rac-(*cis*)-1-Benzyl-3-*tert*-butyl-5-allyl-4-oxo-2-phenyl-dihydropyrimidine-1,3(2*H*,4*H*)-dicarboxylate (*rac*-11c)

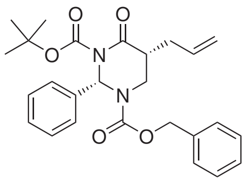

M = 450.53 g mol^{-1} $C_{26}H_{30}N_2O_5$

Reaction of ***rac*-5** and allyl bromide according to GP1 affords ***rac*-11c** (0.20 g, 73 %) as highly viscous colourless oil. *dr* = 95.4 : 4.6 (HPLC). **HPLC** (method M1): t_R = 35.2 min (major diastereomer), 35.5 min (minor diastereomer). ^{1}**H-NMR** (250 MHz, $CDCl_3$, TMS): δ [ppm] = 1.38/1.53 (2 s, 9H, $C(CH_3)_3$), 2.12 (m, 1H, $C\underline{H}^AH^BCH{=}CH_2$), 2.39 (br m, 1H, C^5H), 2.53 (m, 1H, $CH^A\underline{H}^BCH{=}CH_2$), 3.54 (dd, J = 12.2, 10.2 Hz, 1H, $C^6\underline{H}^AH^B$), 3.62 (dd, J = 12.2, 8.0 Hz, 1H, $C^6H^AC\underline{H}^B$), 4.92-5.03 (m, 2H, $CH{=}C\underline{H}_2$), 5.23 (d, J = 12.5 Hz, 1H, $C\underline{H}^AH^BPh$), 5.29 (d, J = 12.3 Hz, 1H, $CH^A\underline{H}^BPh$), 5.64 (dddd, J = 17.5, 9.7, 7.6, 6.3 Hz, 1H, $C\underline{H}{=}CH_2$), 7.17-7.41 (m, 10H, $C^{ar}H$), 7.57 (br s, C^2H). ^{13}**C{^{1}H}-NMR** (63 MHz, $CDCl_3$, TMS): δ [ppm] = 27.9 ($C(\underline{C}H_3)_3$), 32.6 ($\underline{C}H_2CH{=}CH_2$), 40.7/40.8 ($C^5H$), 43.4/43.9 ($C^6H_2$), 67.3 ($C^2H$), 68.0 ($\underline{C}H_2Ph$), 84.2 ($\underline{C}(CH_3)_3$), 117.7 ($CH{=}\underline{C}H_2$), 125.0, 128.1, 128.4, 128.6, 129.2 ($C^{ar}H$), 134.3/134.4 ($\underline{C}H{=}CH_2$), 136.0, 138.4 (C^{ar}), 150.7/151.0, 154.5/155.4 (NCO_2), 170.3/170.4 (C^4). **IR** (film, NaCl): $\tilde{\nu}$ [cm^{-1}] = 2980 m, 1779 m, 1720 vs, 1496 w, 1450 m, 1410 m, 1369 m, 1289 s, 1148 s, 1030 w, 960 w, 918 w, 851 w, 735 m, 697 m. **HRMS** (ESI-FT-ICR): *m/z* = 473.2041 (calculated for $[C_{26}H_{30}N_2O_5+Na]^+$: 473.2052); 923.4193 (calculated for $[(C_{26}H_{30}N_2O_5)_2+Na]^+$: 923.4220).

(2*S*,5*R*)-1-Benzyl-3-*tert*-butyl-5-(2-*tert*-butoxy-2-oxoethyl)-4-oxo-2-phenyldihydropyrimidine-1,3(2*H*,4*H*)-dicarboxylate ((2*S*,5*R*)-11d)

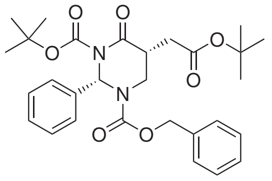

M = 524.61 g mol^{-1} $C_{29}H_{36}N_2O_7$

Reaction of **(*S*)-5** and *tert*-butyl bromoacetate according to GP1 affords **(2*S*,5*R*)-11d** (0.30 g, 94 %) as highly viscous colourless oil. *dr* = 94.6 : 5.4 (HPLC). **HPLC** (method M1): t_R = 36.1 min (major diastereomer), 37.1 min (minor diastereomer). $[\alpha]_D^{RT}$ = + 26.1 (c = 2.15 in $CHCl_3$). ^{1}**H-NMR** (250 MHz, $CDCl_3$, TMS): δ [ppm] = 1.35/1.37 (s, 9H, $C(CH_3)_3$, ester), 1.53 (br s, 9H, $C(CH_3)_3$, Boc), 2.15 (dd, *J* = 16.7, 6.5 Hz, 1H, $C\underline{H}^AH^B$-CO_2t-Bu), 2.71 (dd, *J* = 16.7, 6.1 Hz, 1H, $CH^A\underline{H}^BCO_2t$-Bu), 2.86 (m, 1H, C^5H), 3.57 (dd, *J* = 11.8, 11.5 Hz, 1H, $C^6\underline{H}^AH^B$), 3.71 (dd, *J* = 11.8, 8.0 Hz, 1H, $C^6H^A\underline{H}^B$), 5.22 (d, *J* = 12.4 Hz, 1H, $C\underline{H}^AH^BPh$), 5.29 (d, *J* = 12.3 Hz, 1H, $CH^A\underline{H}^BPh$), 7.21-7.43 (m, 10H, $C^{ar}H$), 7.61 (s, C^2H). ^{13}**C{^{1}H}-NMR** (63 MHz, $CDCl_3$, TMS): δ [ppm] = 27.9 (2 × $C(\underline{C}H_3)_3$), 34.2 ($\underline{C}H_2CO_2t$-Bu), 38.0 (C^5H), 44.0/44.4 (C^6H), 67.4 (C^2H), 68.1 ($\underline{C}H_2Ph$), 80.9 ($\underline{C}(CH_3)_3$, Boc), 84.3 ($\underline{C}(CH_3)_3$, Ester), 124.9, 128.0, 128.4, 128.6, 129.2 ($C^{ar}H$), 135.9, 138.1/138.2 (C^{ar}), 150.5/150.7, 154.4/155.3 (NCO_2), 169.6/169.7, 169.8 (CH_2-$\underline{C}O_2t$-Bu and C^4). **IR** (film, NaCl): $\tilde{\nu}$ [cm^{-1}] = 3064 w, 3034 w, 2980 m, 2933 m, 2254 w, 1780 s, 1730 vs, 1602 w, 1586 w, 1497 w, 1451 m, 1410 s, 1369 s, 1281 s, 1147 s, 1031 w, 970 m, 914 m, 850 m, 793 w, 734 s, 697 m. **HRMS** (ESI-FT-ICR): *m/z* = 547.2411 (calculated for $[C_{29}H_{36}N_2O_7+Na]^+$: 547.2396).

rac-(*cis*)-1-Benzyl-5-benzyl-4-oxo-2-phenyltetrahydropyrimidine-1(2*H*)-carboxylate (*rac*-13)

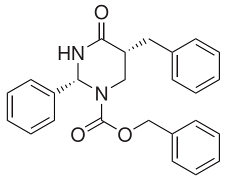

M = 400.47 g mol^{-1} $C_{25}H_{24}N_2O_3$

TFA (7 mL) is added dropwise to an ice cooled solution of ***rac*-11b** (652 mg, 1.30 mmol) in abs. CH_2Cl_2 (100 mL). The reaction solution is stirred for 1 h at 0 °C, all volatile components are evaporated in vacuo and the residue is coevaporated with toluene. Purification by flash chromatography (eluent: EtOAc/petrol ether 3 : 2 v/v) yields ***rac*-13** (400 mg, 77 %) as a colourless, highly viscous oil. ^{1}**H-NMR** (500 MHz, $CDCl_3$, TMS): δ [ppm] = 2.59

(dddd, J = 11.4, 4.6, 2.7, 2.6 Hz, 1H, C^5H), 2.65 (dd, J = 13.0, 10.9 Hz, 1H, $C^6\underline{H}^AH^B$), 3.11 (dd, J = 13.8, 4.4 Hz, 1H, $C^5HC\underline{H}^AH^BPh$), 3.18 (dd, J = 13.6, 2.6 Hz, 1H, $C^5HCH^A\underline{H}^BPh$), 3.83 (m, 1H, $C^6H^A\underline{H}^B$), 5.20 (d, J = 11.9 Hz, 1H, $OC\underline{H}^AH^BPh$), 5.29 (d, J = 11.9 Hz, 1H, $OCH^A\underline{H}^BPh$), 6.68 (s, 1H, C^2H), 7.04-7.40 (m, 15H, $C^{ar}H$), 7.52 (s, 1H, NH). **$^{13}C\{^1H\}$-NMR** (126 MHz, $CDCl_3$, TMS): δ [ppm] = 36.1 ($C^5H\underline{C}H_2Ph$), 38.7 (C^5H), 43.3 (C^6H_2), 65.4 (C^2H), 68.2 ($O\underline{C}H_2Ph$), 126.1, 126.5, 126.6, 128.4, 128.5, 128.6, 128.7, 128.8, 129.2 ($C^{ar}H$), 135.7, 138.5, 138.9 (C^{ar}), 154.8 (NCO_2), 173.0 (C^4). **MS** (ESI, cation mode): *m/z* = 401.2 $[M+Na]^+$ (calculated for $[C_{25}H_{25}N_2O_2]^+$: 401.2), 423.2 $[M+Na]^+$ (calculated for $[C_{25}H_{24}N_2O_3+Na]^+$: 423.2).

Ring cleavage and isolation of β^2-amino acid

Fmoc-(*R*)-β^2-hAsp(O*t*-Bu)-OH (16)

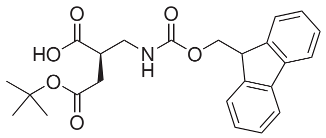

M = 425.47 g mol^{-1} $C_{24}H_{27}NO_6$

An ice cooled solution of **(2*S*,5*R*)-11d** (43 mg, 0.082 mmol) in THF (1.5 mL) is treated with 30 % aq. H_2O_2 (0.04 mL, 0.39 mmol H_2O_2, 5.0 equiv.), followed by slow addition of $LiOH \cdot H_2O$ (7.6 mg, 0.18 mmol, 2.2 equiv.) as solution in H_2O (0.3 mL). The reaction mixture is slowly warmed to rt upon stirring for 5 h. After addition of 5 % aq. Na_2SO_3 and 5 % aq. $NaHCO_3$ (2 mL each), the mixture is stirred for 20 min at rt. The containing THF is evaporated, the aqueous residue is diluted with H_2O (10 mL), acidified by addition of 5 % aq. $KHSO_4$ and extracted with CH_2Cl_2 (6 × 15 mL). The combined organic extracts are dried over $MgSO_4$, evaporated to dryness, and dried in vacuo. Ring opening product **15** is obtained as a colourless highly viscous oil. **MS** (ESI, cation mode): *m/z* = 542.8 $[M+H]^+$ (calculated: 543.3); **MS** (ESI, anion mode): *m/z* = 541.1 $[M–H]^-$ (calculated: 541.3), 577.2 $[M+Cl]^-$ (calculated: 577.2).

Pd/C-catalyst (10 % Pd, 10 mg) is added to a solution of crude **15** (44 mg) in MeOH (3 mL). The mixture is deoxygenated by purging with argon and subsequently stirred over night at rt under H_2 atmosphere (1 bar, balloon). Excessive H_2 is driven out with argon, the reaction mixture is filtered and evaporated to dryness. The crude deprotected β^2-amino acid is taken up in H_2O (1 mL). $NaHCO_3$ (20 mg, 0.24 mmol, 3 equiv.), FmocOSu (30 mg, 0.08 mmol, 1.1 equiv.) and acetone (1 mL) are subsequently added. The reaction mixture is stirred for 4 h at rt, concentrated to a volume of ca. 1 mL and diluted with saturated aq. $NaHCO_3$ (15 mL). The aqueous mixture is extracted with Et_2O (3 × 15 mL) and subsequently acidified by addition of 5 % aq. $KHSO_4$. The acidic solution is extracted with

EtOAc (3 × 50 mL), the combined EtOAc extracts are dried over $MgSO_4$ and evaporated to dryness. Purification by column chromatography (eluent: CH_2Cl_2 + 1 vol.-% AcOH), followed by lyophilisation affords **16** (23 mg, 67 %) as a colourless solid. $[\alpha]_D^{RT} = -1.2$ [c = 1.00 in $CHCl_3$ (ref. [5] ***ent*-16**: + 1.4 , c = 1.20 in $CHCl_3$)]. ^{1}**H-NMR** (500 MHz, $CDCl_3$, TMS): δ [ppm] = 1.42/1.44 (2s, 9H, $C(CH_3)_3$), 2.38 (dd, J = 16.9, 5.8 Hz, 0.2H, $C\underline{H}^AH^BCO_2t$-Bu), 2.46 (dd, J = 16.6, 7.6 Hz, 0.2H, $CH^A\underline{H}^BCO_2t$-Bu), 2.52 (dd, J = 16.8, 5.2 Hz, 0.8H, $C\underline{H}^AH^BCO_2t$-Bu), 2.64 (dd, J = 16.8, 7.7 Hz, 0.8H, $CH^A\underline{H}^BCO_2t$-Bu), 2.77 (m, 0.2H, $C^{\alpha}H$), 3.00 (m, 0.8H, $C^{\alpha}H$), 3.29 (m, 0.4H, $C\underline{H_2}NH$), 3.43-3.52 (m, 1.6H, $C\underline{H_2}NH$), 4.19 (t, J = 6.9 Hz, 0.8H, $C\underline{H}CH_2$, Fmoc), 4.24 (m, 0.2H, $C\underline{H}CH_2$, Fmoc), 4.37 (d, J = 7.0 Hz, 1.5H, CH_2, Fmoc), 4.48 (m, 0.4H, CH_2, Fmoc), 5.36 (m, 0.8H, NH), 6.15 (m, 0.2H, NH), 7.30 (t, J = 7.5 Hz, 2H, $C^{ar}H$), 7.38 (t, J = 7.4 Hz, 2H, $C^{ar}H$), 7.57 (d, J = 7.5 Hz, 2H, $C^{ar}H$), 7.75 (d, J = 7.5 Hz, 2H, $C^{ar}H$), 8.5 (br s, 1H, CO_2H). ^{13}**C{^{1}H}-NMR** (126 MHz, $CDCl_3$, TMS): δ [ppm] = 28.0 ($C(\underline{C}H_3)_3$), 34.9 ($\underline{C}H_2CO_2t$-Bu), 41.7 ($\underline{C}H_2NH$), 41.8 ($C^{\alpha}H$), 47.2 ($CH_2\underline{C}H$, Fmoc), 66.9 (CH_2, Fmoc), 81.7 ($\underline{C}(CH_3)_3$), 120.0, 124.9/125.1, 127.1/127.2, 127.7/127.8 ($C^{ar}H$), 141.3, 143.8 (C^{ar}), 156.6 ($NHCO_2$), 171.2 ($\underline{C}O_2t$Bu), 177.9 (CO_2H). **IR** (disk, KBr): $\tilde{\nu}$ [cm^{-1}] = 3382 w, 2978 w, 1742 vs, 1525 w, 1450 w, 1368 w, 1259 s, 1151 m, 1151 m. **MS** (ESI, cation mode): *m/z* = 448.2 $[M+Na]^+$ (calculated for $[C_{24}H_{27}NO_6+Na]^+$: 448.2). **MS** (ESI, anion mode): *m/z* = 424.0 $[M–H]^-$ (calculated for $[C_{24}H_{26}NO_6]^-$: 424.2), 460.0 $[M+Cl]^-$ (calculated for $[C_{24}H_{27}NO_6+Cl]^-$: 460.2).

Spectra

(*R*)-4: ^{1}H- and ^{13}C-NMR-spectrum

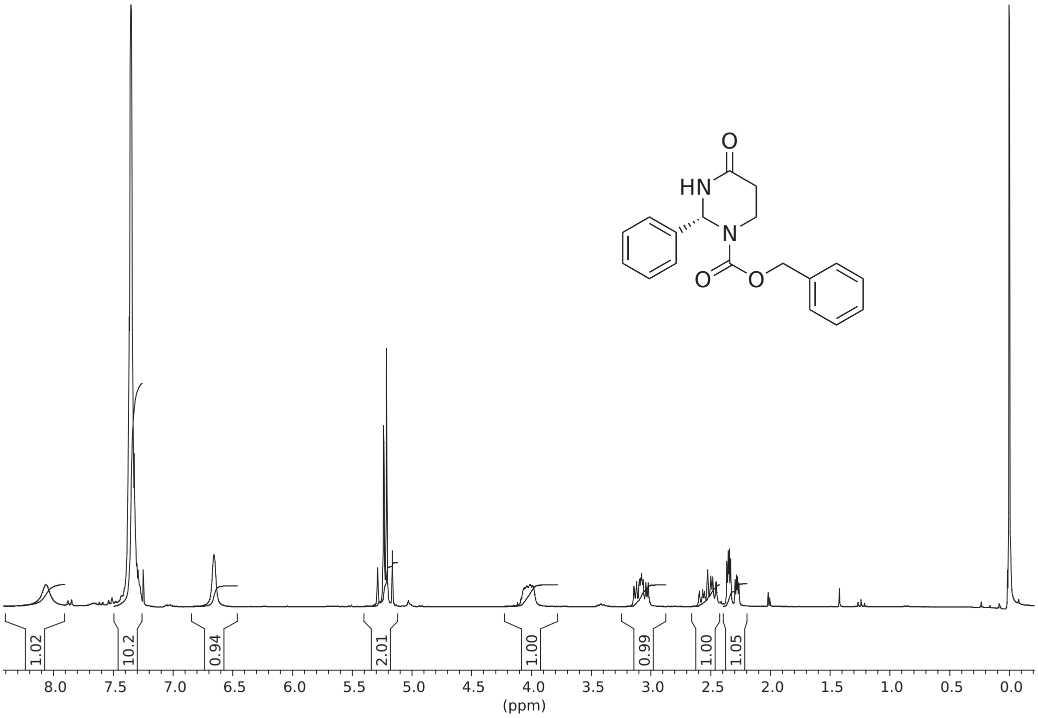

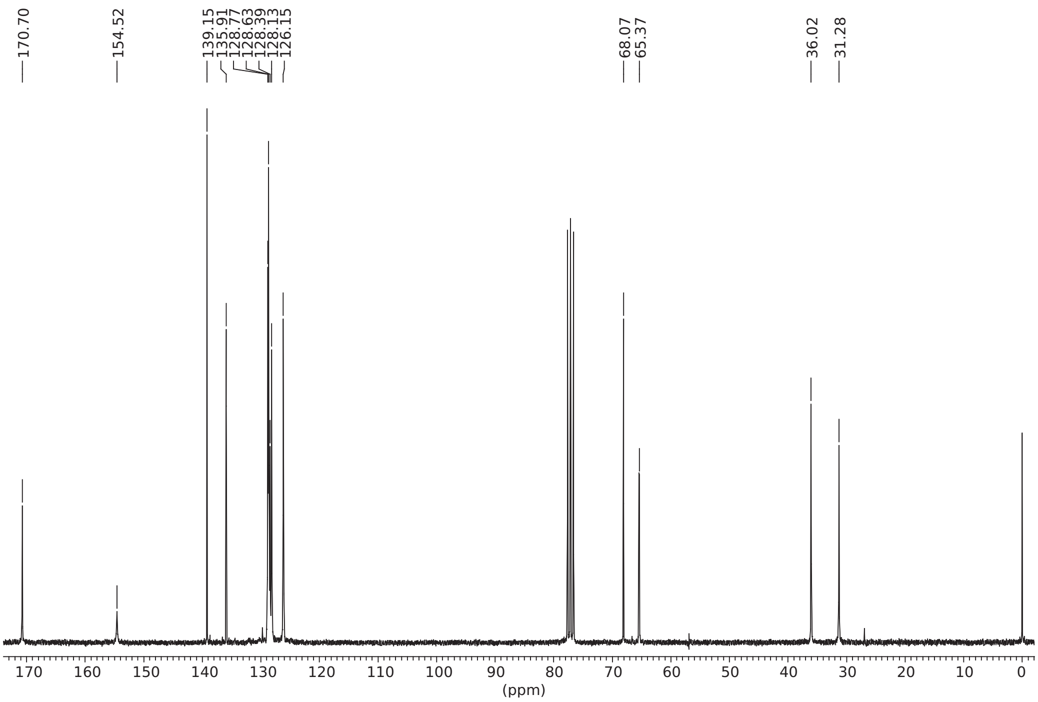

(*S*)-5: ^{1}H- and ^{13}C-NMR-spectrum

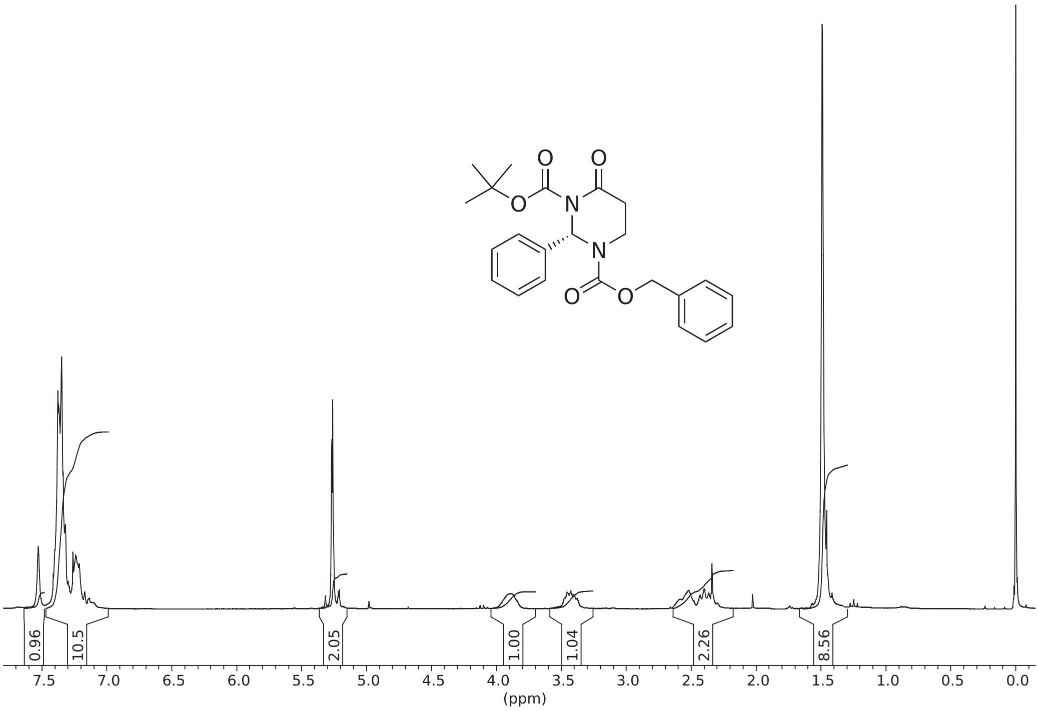

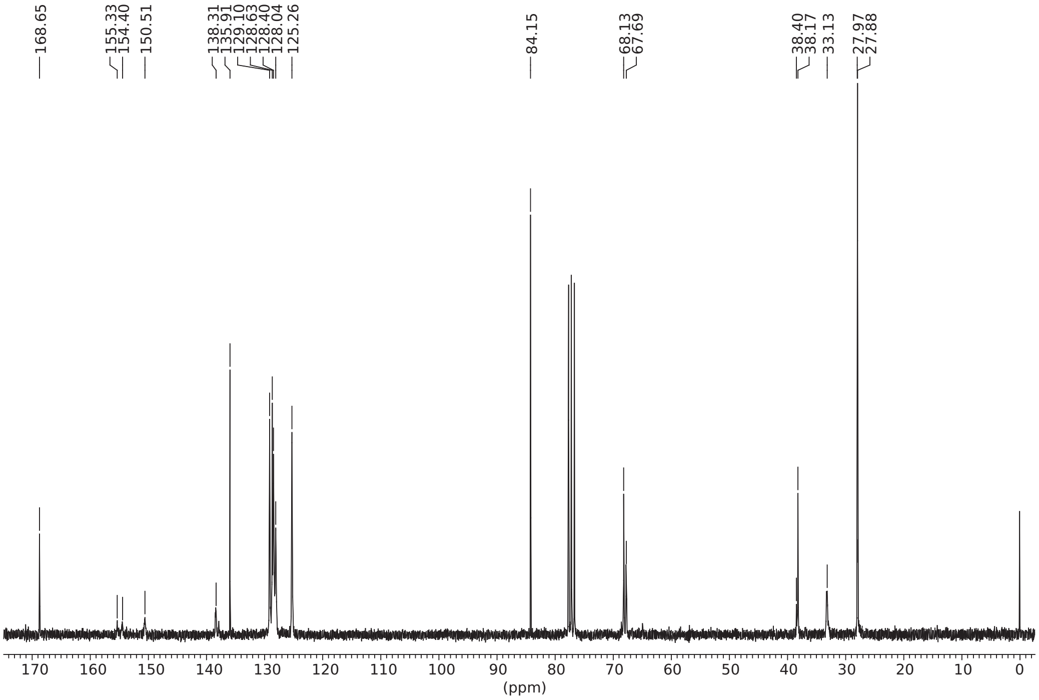

rac-**7**: ^{1}H- and ^{13}C-NMR-spectrum

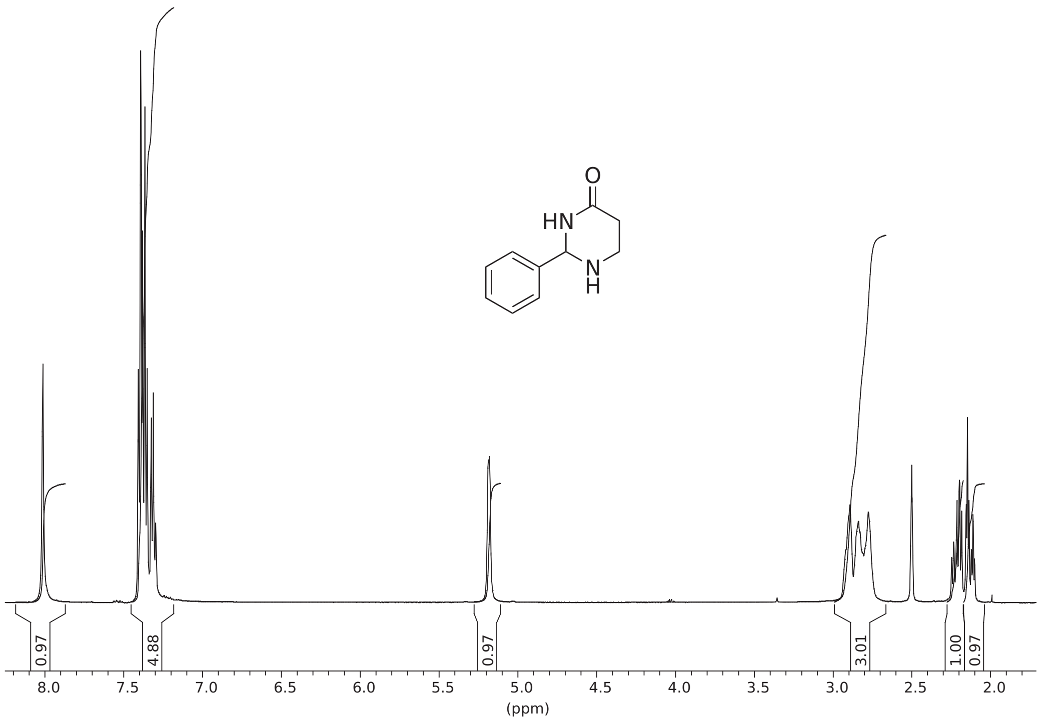

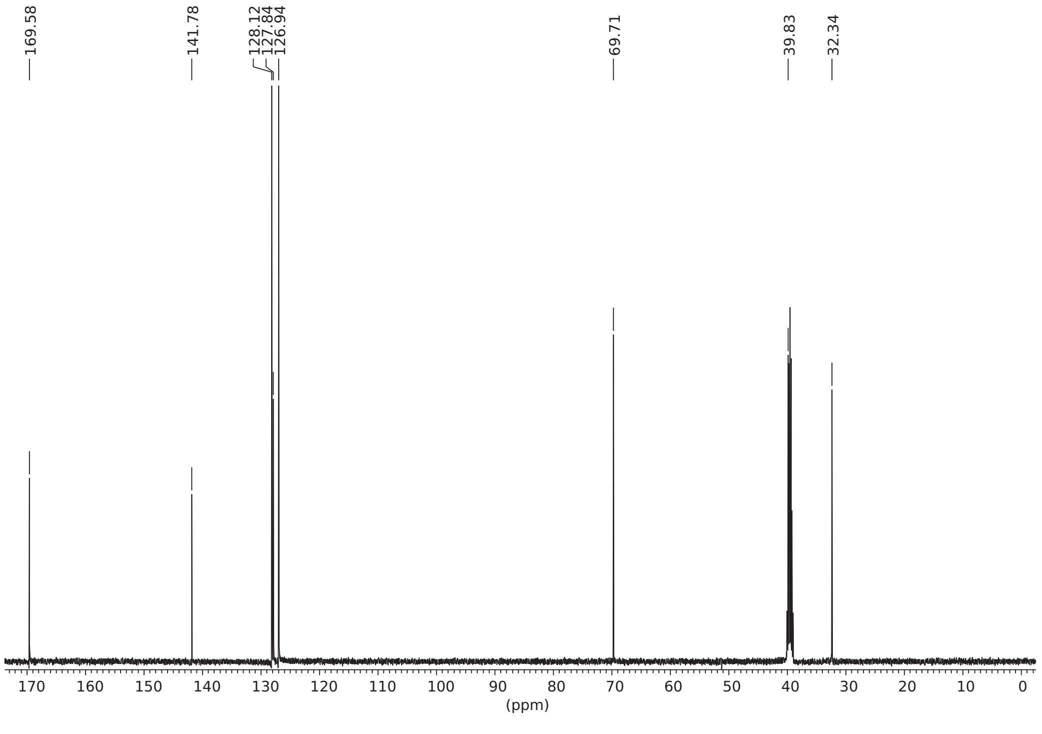

(2*R*,6*S*)- and (2*S*,6*S*)-9: ^{1}H-NMR-spectra

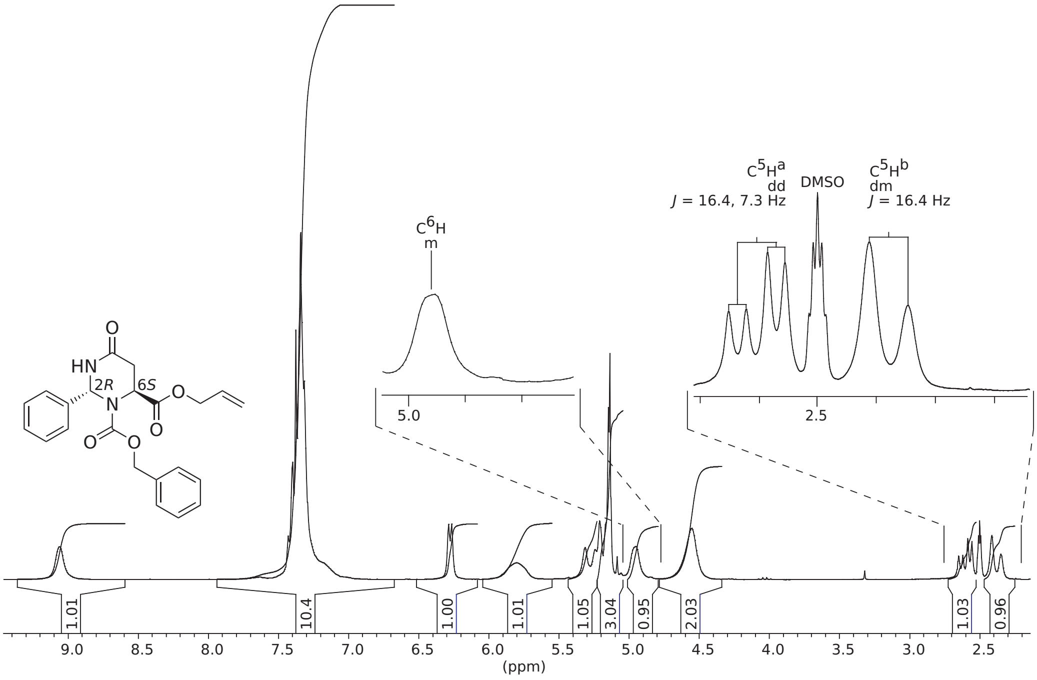

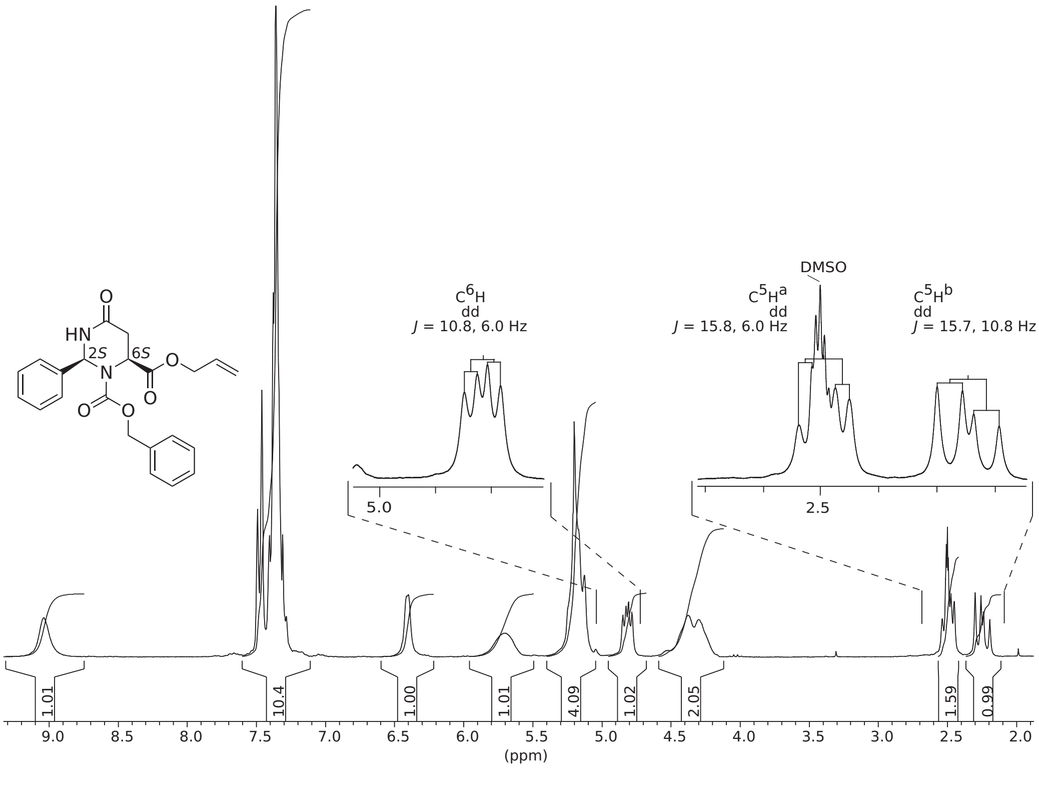

(2*R*,6*S*)- and (2*S*,6*S*)-9: ^{13}C-NMR-spectra

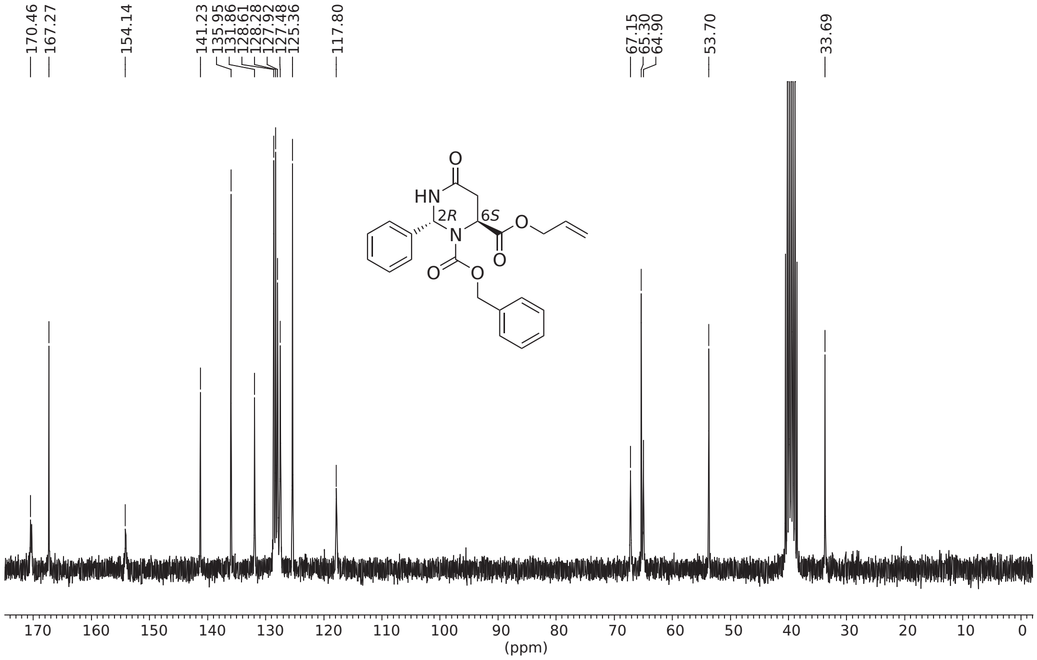

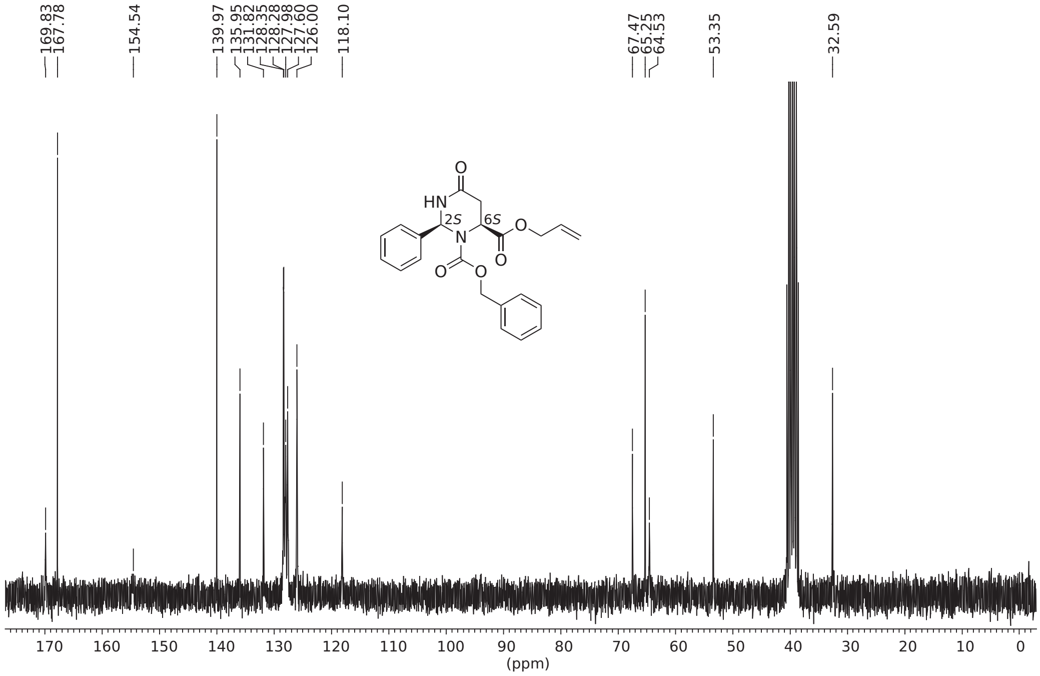

(*R*)-10: ^{1}H- and ^{13}C-NMR-spectrum

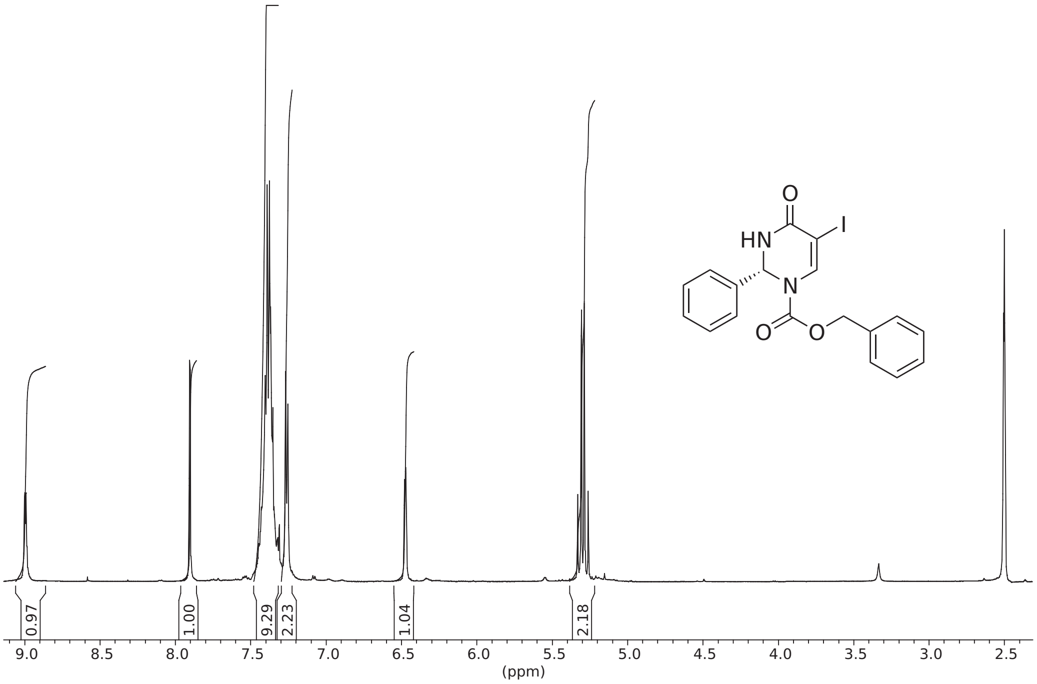

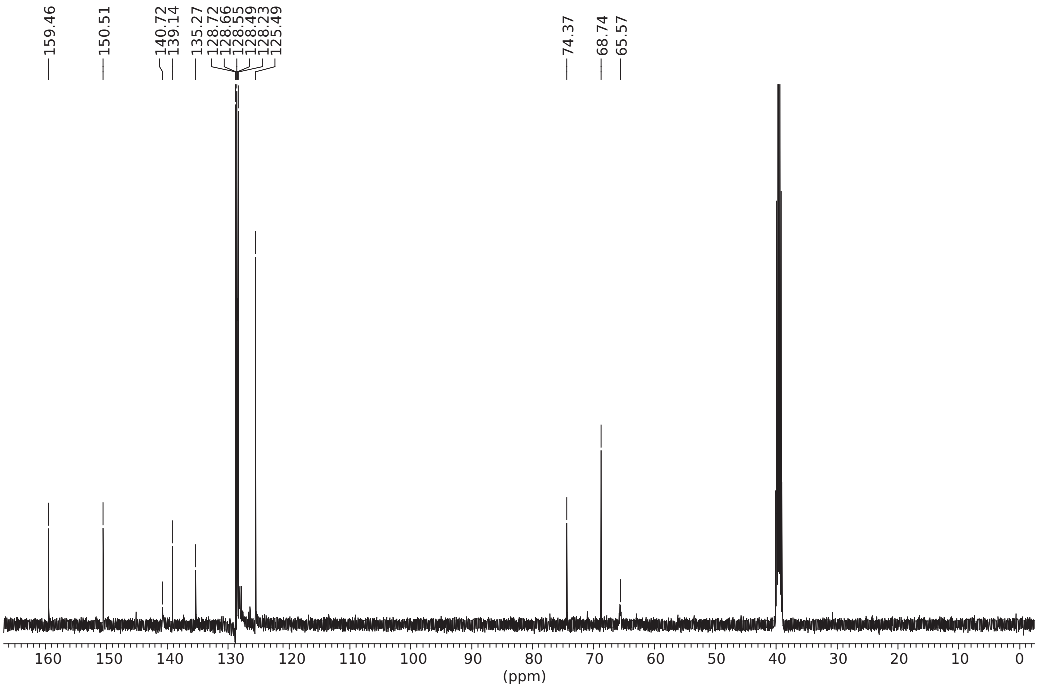

rac-(*cis*)-11a: ^{1}H- and ^{13}C-NMR-spectrum

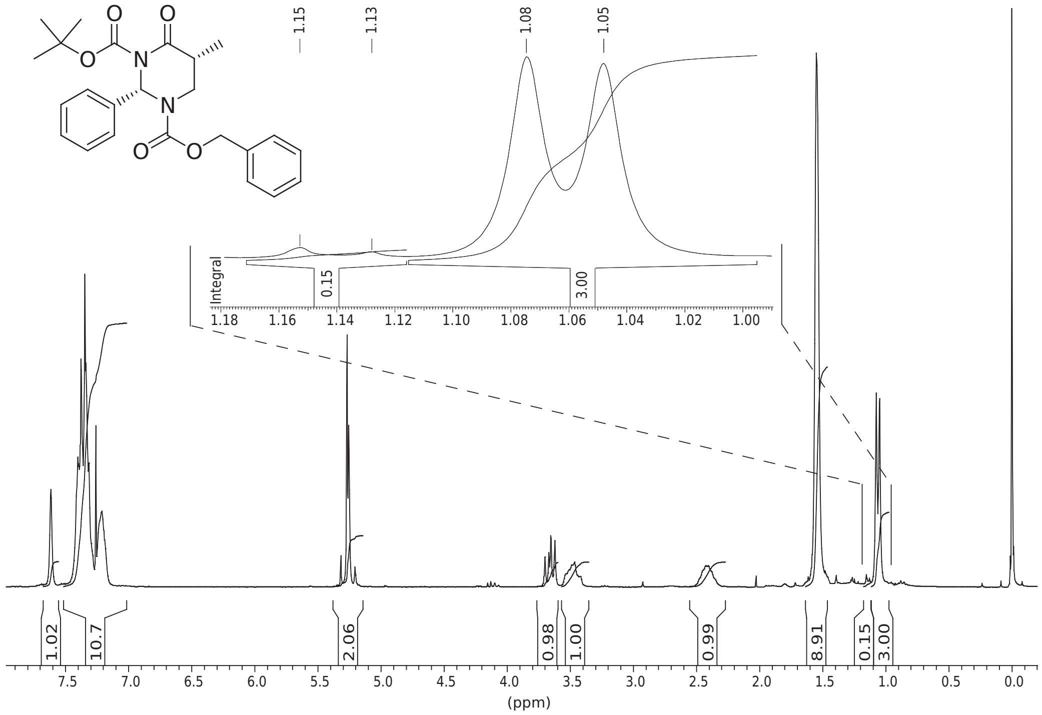

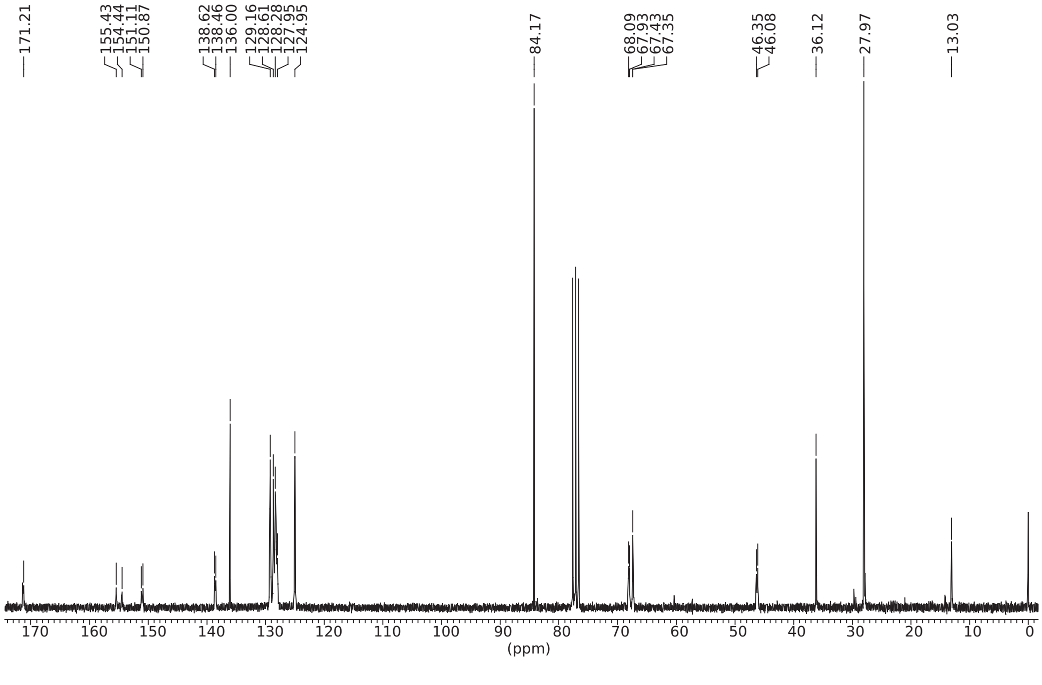

rac-(*cis*)-11b: ^{1}H- and ^{13}C-NMR-spectrum

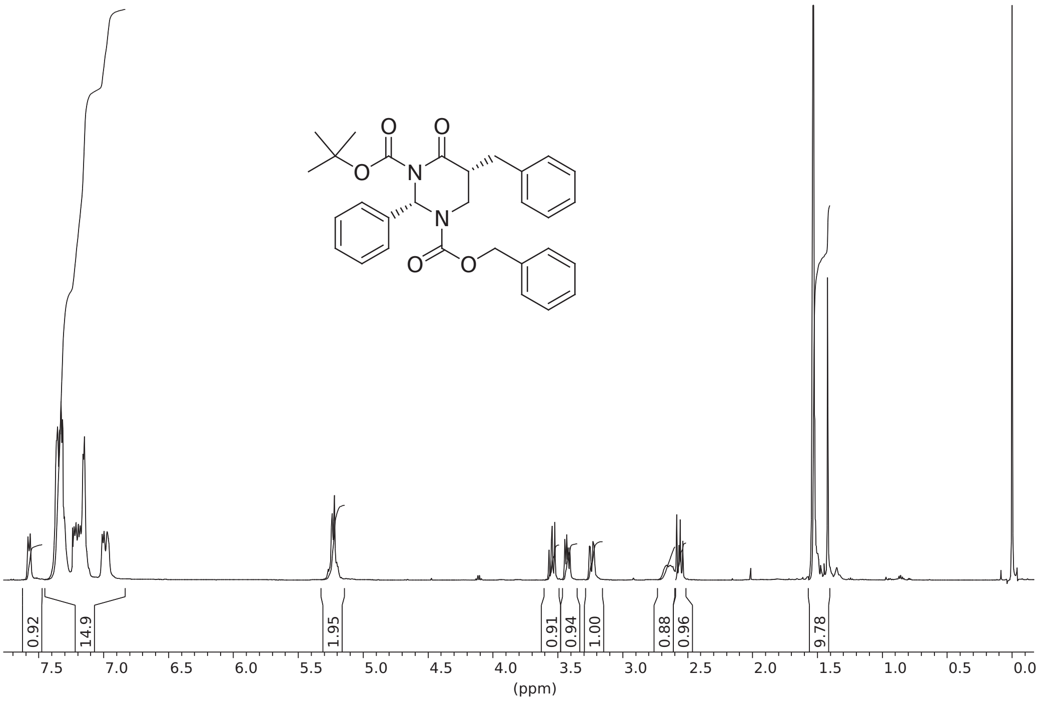

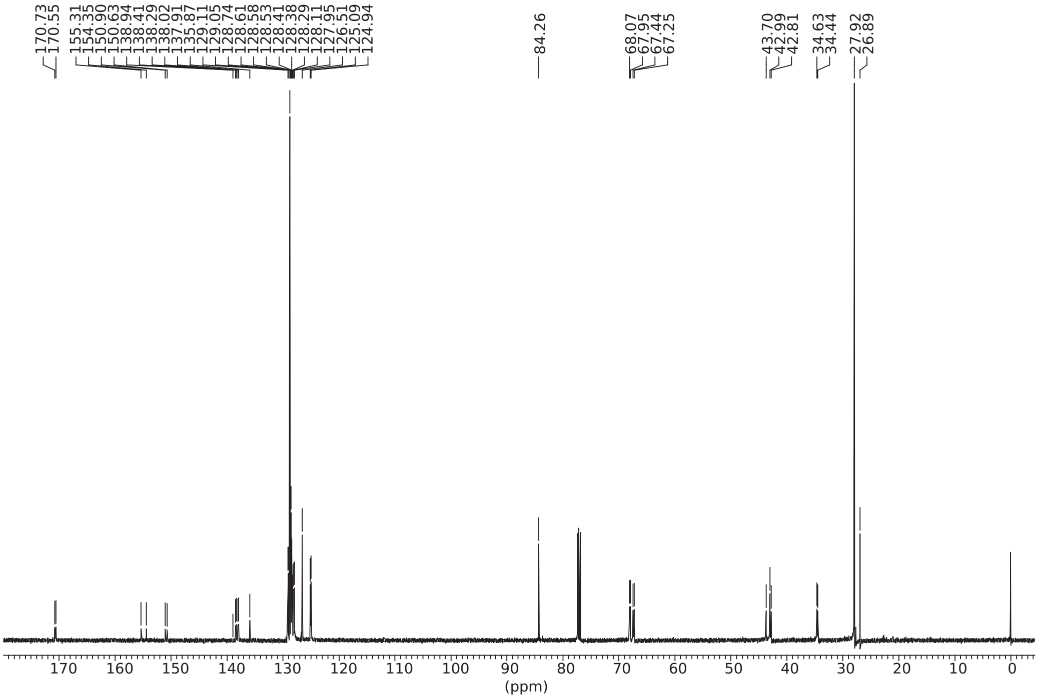

rac-(*cis*)-11c: ^{1}H- and ^{13}C-NMR-spectrum

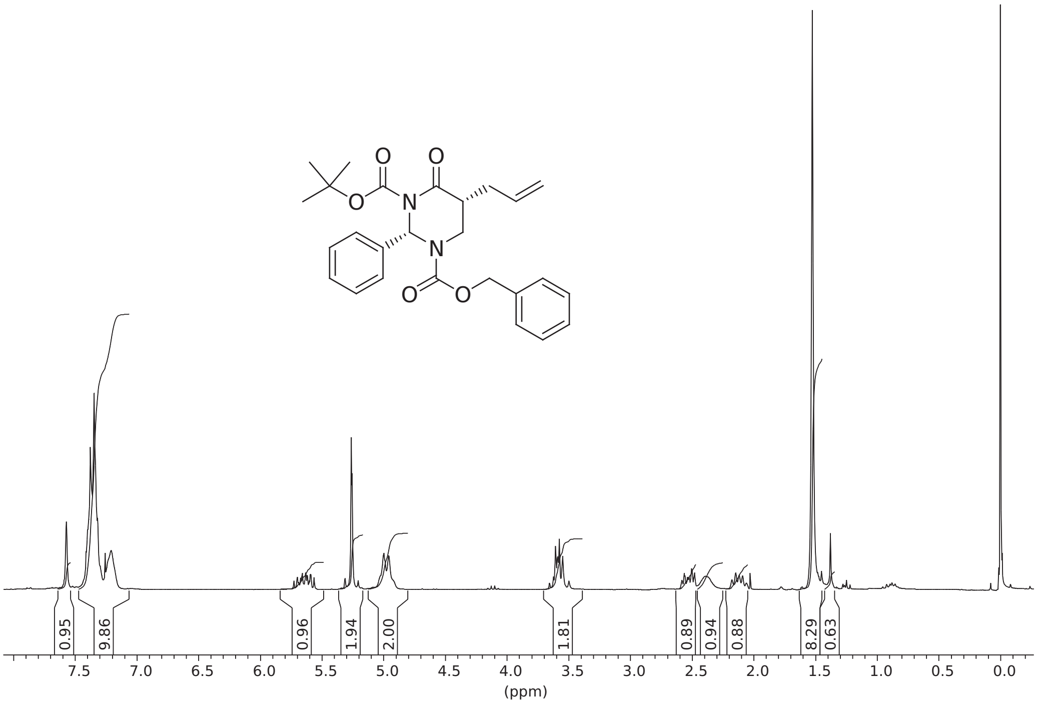

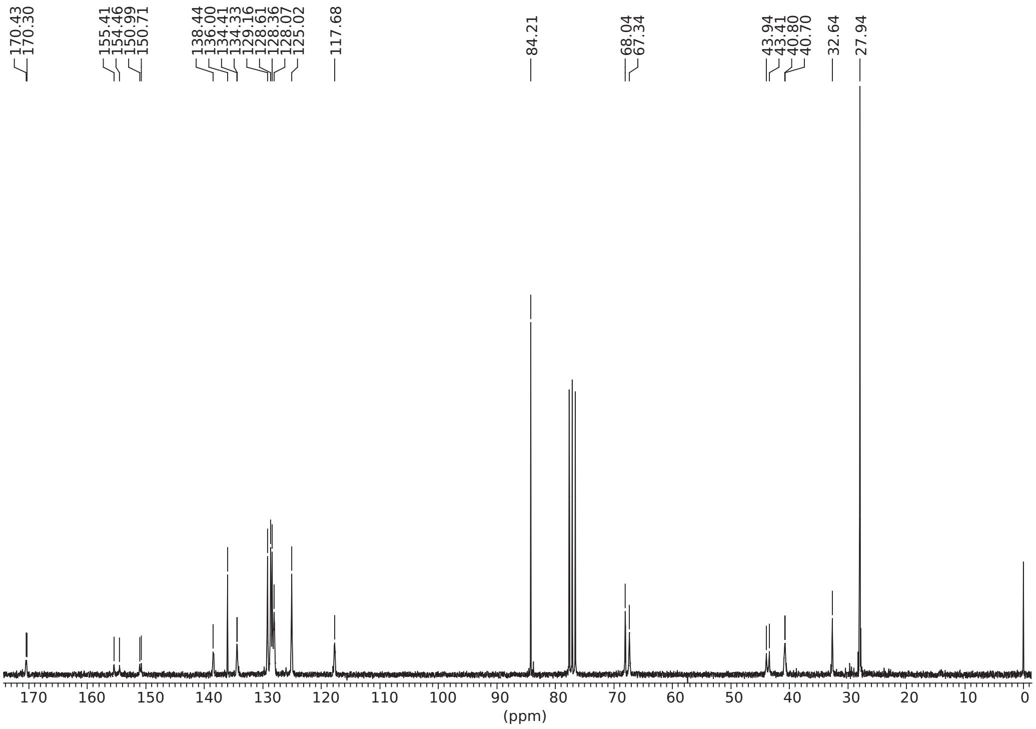

(2*S*,5*R*)-11d: ^{1}H- and ^{13}C-NMR-spectrum

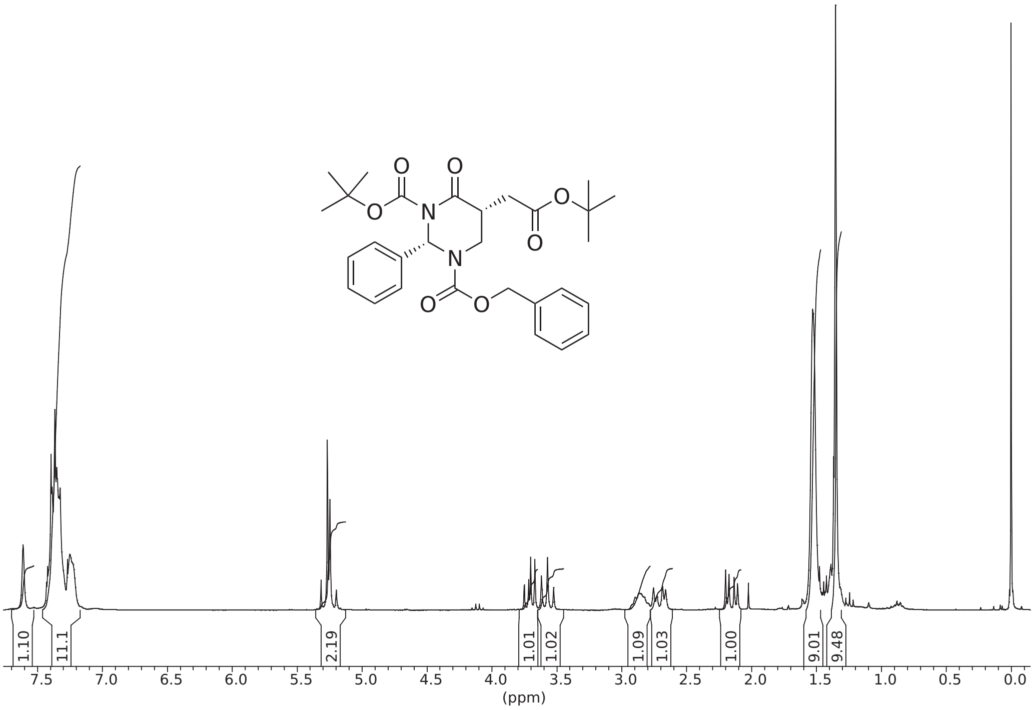

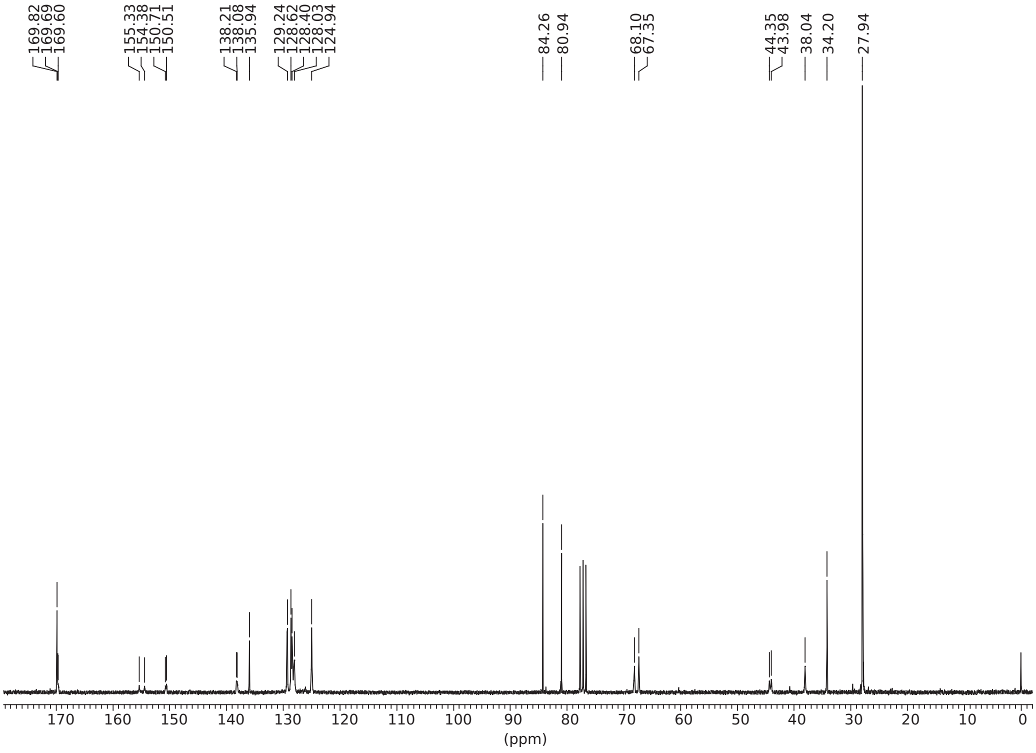

Analytical RP-HPLC of 11b-d

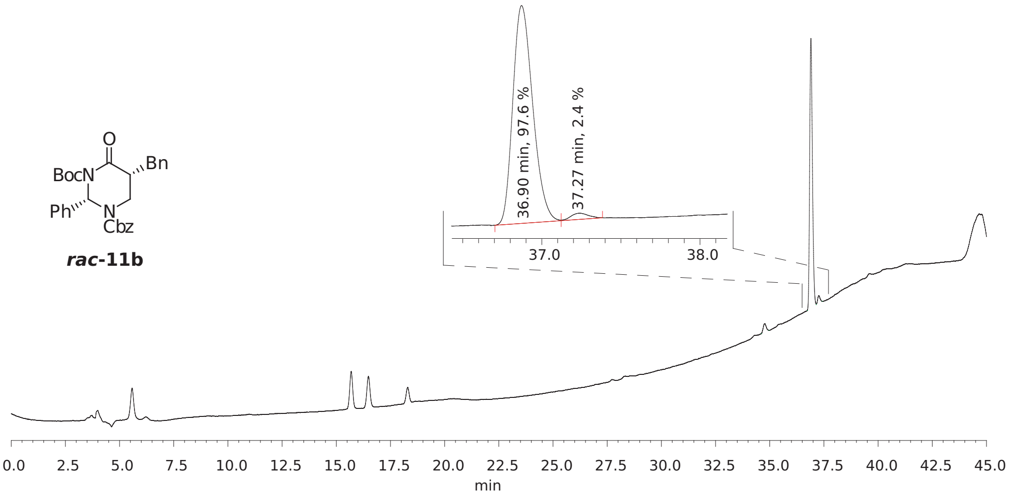

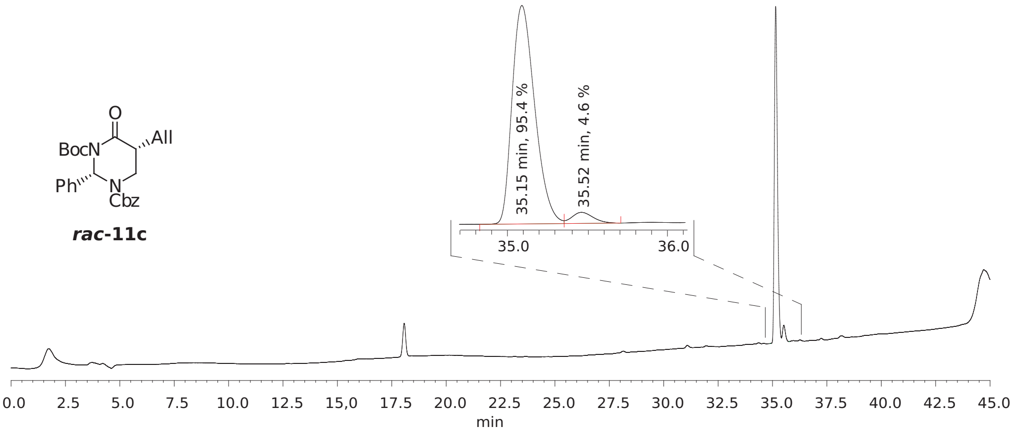

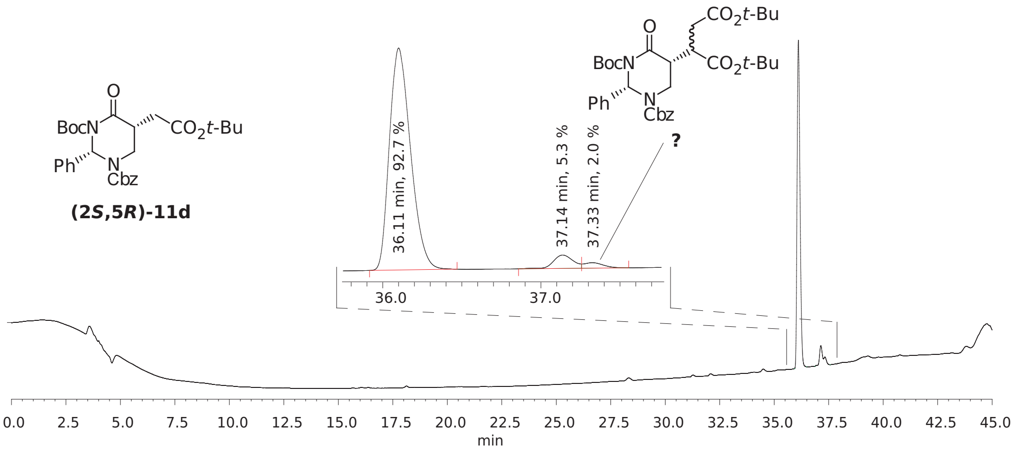

Analytical HPLC plots of crude products **11b-d** (UV-absorption at λ = 254 nm). No residual starting material **5** (t_R = 31.3 min) was detectable.

^{1}H- and ^{13}C-NMR-spectrum of Fmoc-(*R*)-β^2-hAsp(O*t*-Bu)-OH (16)

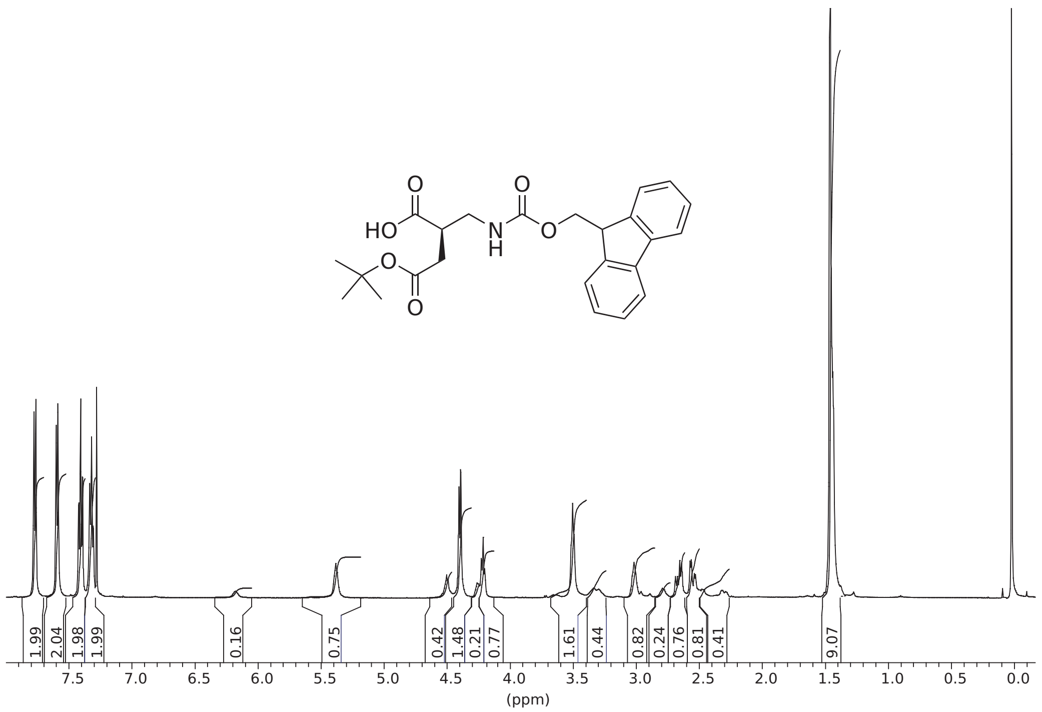

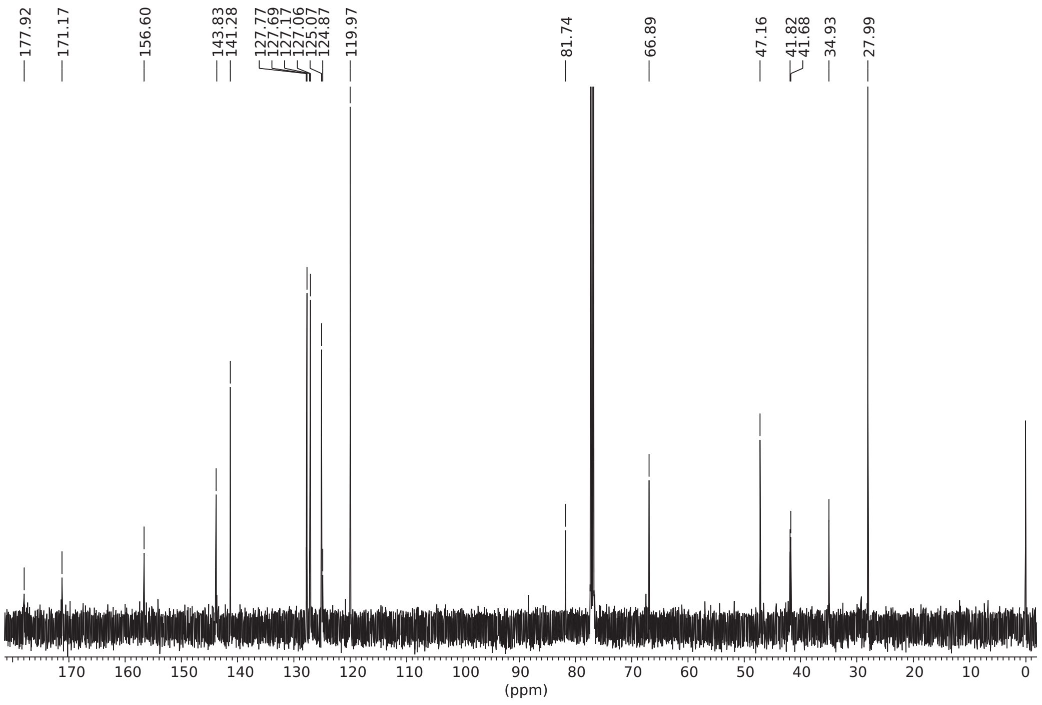

Bibliography

[1] Gottlieb, H. E.; Kotlyar, V.; Nudelman, A. *J. Org. Chem.* **1997,** *62*, 7512-7515.

[2] Kricheldorf, H. R.; Schwarz, G.; Kaschig, J. *Angew. Chem.* **1977,** *89,* 570-572; *Angew. Chem., Int. Ed.* **1977**, *16*, 550-552.

[3] Hanson, H. T.; Smith, E. L. *J. Biol. Chem.* **1948,** *175*, 833-848.

[4] Diaz-Sanchez, B. R.; Iglesias-Arteaga, M. A.; Melgar-Fernandez, R.; Juaristi, E. *J. Org. Chem.* **2007,** *72,* 4822-4825.

[5] Lelais, G.; Campo, M. A.; Kopp, S.; Seebach, D. *Helv. Chim. Acta* **2004,** *87,* 1545-1559.
